# Completing the is-a structure in light-weight ontologies

**DOI:** 10.1186/s13326-015-0002-8

**Published:** 2015-03-28

**Authors:** Patrick Lambrix, Fang Wei-Kleiner, Zlatan Dragisic

**Affiliations:** Department of Computer and Information Science, Linköping University, Linköping, Sweden; Swedish e-Science Research Centre, Linköping University, Linköping, Sweden

**Keywords:** Ontologies, Ontology engineering, Ontology debugging

## Abstract

**Background:**

With the increasing presence of biomedical data sources on the Internet more and more research effort is put into finding possible ways for integrating and searching such often heterogeneous sources. Ontologies are a key technology in this effort. However, developing ontologies is not an easy task and often the resulting ontologies are not complete. In addition to being problematic for the correct modelling of a domain, such incomplete ontologies, when used in semantically-enabled applications, can lead to valid conclusions being missed.

**Results:**

We consider the problem of repairing missing is-a relations in ontologies. We formalize the problem as a generalized TBox abduction problem. Based on this abduction framework, we present complexity results for the existence, relevance and necessity decision problems for the generalized TBox abduction problem with and without some specific preference relations for ontologies that can be represented using a member of the ${\mathcal {EL}}$ family of description logics. Further, we present algorithms for finding solutions, a system as well as experiments.

**Conclusions:**

Semantically-enabled applications need high quality ontologies and one key aspect is their completeness. We have introduced a framework and system that provides an environment for supporting domain experts to complete the is-a structure of ontologies. We have shown the usefulness of the approach in different experiments. For the two Anatomy ontologies from the Ontology Alignment Evaluation Initiative, we repaired 94 and 58 initial given missing is-a relations, respectively, and detected and repaired additionally, 47 and 10 missing is-a relations. In an experiment with BioTop without given missing is-a relations, we detected and repaired 40 new missing is-a relations.

## Background

With the increasing presence of biomedical data sources on the Internet more and more research effort is put into finding possible ways for integrating and searching such often heterogeneous sources. Semantic Web technologies such as ontologies, are becoming a key technology in this effort. Ontologies provide a means for modelling the domain of interest and they allow for information reuse, portability and sharing across multiple platforms. Efforts such as the Open Biological and Biomedical Ontologies (OBO) Foundry [1], BioPortal [2] and Unified Medical Language System (UMLS) [3] aim at providing repositories for biomedical ontologies and relations between these ontologies thus providing means for annotating and sharing biomedical data sources. Many of the ontologies in the biomedical domain, e.g., SNOMED [4] and Gene Ontology [5], are, regarding knowledge representation, light-weight ontologies. They are taxonomies or can be represented using the ${\mathcal {EL}}$ description logic or small extensions thereof (e.g. [6] and the TONES Ontology Repository [7])^a^. Therefore, in this paper, we consider ontologies that are represented by TBoxes in the ${\mathcal {EL}}$ family, which consist of axioms such as *Carditis*$\sqsubseteq $*Fracture*, with the intended meaning that *Carditis* is a *Fracture*, where *Carditis* and *Fracture* are *concepts* and the relationship is an *is-a* relation. (For detailed syntax see Section [Sec Sec3]). A set of such terminological axioms is a TBox.

Developing ontologies is not an easy task and often the resulting ontologies (including their is-a structures) are not complete. In addition to being problematic for the correct modelling of a domain, such incomplete ontologies also influence the quality of semantically-enabled applications. Incomplete ontologies when used in semantically-enabled applications can lead to valid conclusions being missed. For instance, in ontology-based search, queries are refined and expanded by moving up and down the hierarchy of concepts. Incomplete structure in ontologies influences the quality of the search results. As an example, suppose we want to find articles in PubMed [8] using the MeSH [9] term *Scleral Disease*. By default the query will follow the hierarchy of MeSH and include more specific terms for searching, such as *Scleritis*. If the relation between *Scleral Disease* and *Scleritis* is missing in MeSH, we will miss 922 articles in the search result, which is about 57% of the original result^b^. The structural information is also important information in ontology engineering research. For instance, most current ontology alignment systems use structure-based strategies to find mappings between the terms in different ontologies (e.g. overview in [10]) and the modeling defects in the structure of the ontologies have an important influence on the quality of the ontology alignment results.

**Figure 1:**
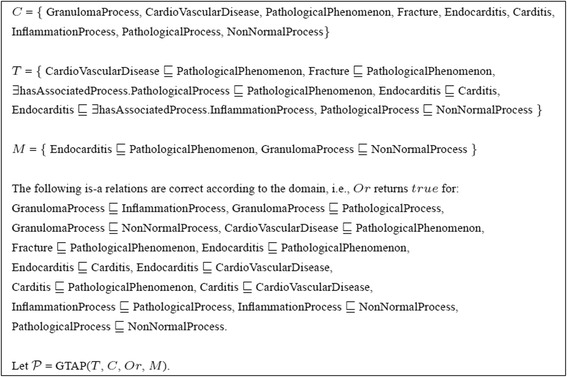
Small ${\mathcal {EL}}$ example. (C is the set of atomic concepts in the ontology. T is a TBox representing the ontology. M is a set of missing is-a relations. Or is the oracle representing the domain expert).

**Figure 2:**
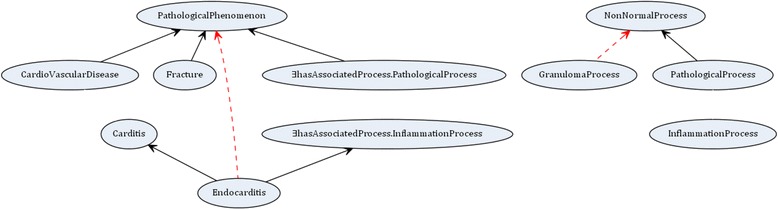
Graphical representation of the ${\mathcal {EL}}$ example in Figure 1. (Ovals represent concepts. Full arrows represent is-a relations between concepts in the ontology. Dashed arrows represent missing is-a relations).

In this paper we tackle the problem of completing the is-a structure of ontologies. Completing the is-a structure requires adding new correct is-a relations to the ontology. We identify two cases for finding relations which need to be added to an ontology. In **case 1** missing is-a relations have been detected and the task is to find ways of making these detected is-a relations derivable in the ontology. There are many approaches to detect missing is-a relations, e.g., in ontology learning [11] or evolution [12], using linguistic [13] and logical [14,15] patterns, by using knowledge intrinsic to an ontology network [16-21], or by using machine learning and statistical methods [22-26]. However, in general, these approaches do not detect *all* missing is-a relations and in several cases even only few. Therefore, we assume that we have obtained a set of missing is-a relations for a given ontology (but not necessarily all). In the case where our set of missing is-a relations contains *all* missing is-a relations, completing the ontology is easy. We just add all missing is-a relations to the ontology and a reasoner can compute all logical consequences. However, when the set of missing is-a relations does not contain all missing is-a relations - and this is the common case - there are different ways to complete the ontology. The easiest way is still to just add the missing is-a relations to the ontology. For instance, *T* in Figure 1 (and Figure 2) represents a small ontology inspired by Galen ontology (http://www.openclinical.org/prj_galen.html), that is relevant for our discussions. Assume that we have detected that Endocarditis $\sqsubseteq $ PathologicalPhenomenon and GranulomaProcess $\sqsubseteq $ NonNormalProcess are missing is-a relations (*M* in Figure 1). Obviously, adding these relations to the ontology will repair the missing is-a structure. However, there are other more interesting possibilities. For instance, adding Carditis $\sqsubseteq $ CardioVascularDisease and GranulomaProcess $\sqsubseteq $ PathologicalProcess also repairs the missing is-a structure. Further, these is-a relations are correct according to the domain and constitute new is-a relations (e.g. Carditis $\sqsubseteq $ CardioVascularDisease) that were not derivable from the ontology and not originally detected by the detection algorithm^c^. We also note that from a logical point of view, adding Carditis $\sqsubseteq $ Fracture and GranulomaProcess $\sqsubseteq $ NonNormalProcess also repairs the missing is-a structure. However, from the point of view of the domain, this solution is not correct. Therefore, as it is the case for all approaches for dealing with modeling defects, a domain expert needs to validate the logical solutions.

In **case 2** no missing is-a relations are given. In this case we investigate existing is-a relations in the ontology and try to find new ways of deriving these existing is-a relations. This might pinpoint to the necessity of adding new missing is-a relations to the ontology. As an example, let us assume that our ontology contains relations *T*∪*M* in Figure 1. If we assume now that we want to investigate new ways of deriving relations in *M* then obviously adding Carditis $\sqsubseteq $ CardioVascularDisease and GranulomaProcess $\sqsubseteq $ PathologicalProcess would be one possibility given that both are correct according to the domain.

The basic problem underlying the two cases can be formalized in the same way as a new kind of abduction problem (formal definitions in Section Abduction framework). Abduction is a reasoning method to generate explanations for observed symptoms and manifestations. When the application domain is described by a logical theory, it is called *logic-based abduction* [27]. Logic-based abduction is widely applied in diagnosis, planning, and database updates [28], among others. Further, as we have seen above, there may be different ways to complete the is-a structure of ontologies. Therefore, we propose two preference criteria on the solutions for this new abduction problem as well as different ways to combine them and conduct complexity analysis on important decision problems regarding the various preference criteria for ontologies represented using ${\mathcal {EL}}$ or ${\mathcal {EL}^{++}}$.

The contributions of this paper are the following.

- We formalize the repairing of the missing is-a structure in an ontology as a generalized version of the TBox abduction problem (GTAP).
- We present complexity results for the existence, relevance and necessity decision problems for GTAP in ontologies represented in ${\mathcal {EL}}$ and ${\mathcal {EL}^{++}}$ with and without the preference relations subset minimality and semantic maximality as well as three ways of combining these (maxmin, minmax, skyline). Subset minimality is a preference criterion that is often used in abductive reasoning problems. Semantic maximality is a new criterion that is important for GTAP.
- We provide algorithms for finding a skyline optimal solution to GTAP in ontologies represented in ${\mathcal {EL}}$ and ${\mathcal {EL}^{++}}$. Although in theory, maxmin optimal solutions are normally preferred, in practice, they cannot be guaranteed and skyline optimal solutions are the best we can do.
- We provide a system and show its usefulness through experiments.

## Methods

### Preliminaries - description logics ${\mathcal {EL}}$ and ${\mathcal {EL}^{++}}$

Description logics are knowledge representation languages. In description logics concept descriptions are constructed inductively from a set *N*_*C*_ of atomic concepts and a set *N*_*R*_ of atomic roles and (possibly) a set *N*_*I*_ of individual names. The concept constructors for ${\mathcal {EL}^{++}}$ are the top concept ⊤, the bottom concept ⊥, nominals, conjunction, existential restriction and a restricted form of concrete domains. In this paper, we consider the version of ${\mathcal {EL}^{++}}$ without concrete domains. Note that this simplification does not affect the complexity results presented later on. For the syntax of the different constructors see Table 1.

**Table 1:** $\boldsymbol{\mathcal {EL}}^{++}$ **syntax and semantics**

**Name**	**Syntax**	**Semantics**
Top	⊤	$\Delta ^{{\mathcal {I}}}$
Bottom	⊥	*∅*
Nominal	{*a*}	$\{a^{{\mathcal {I}}}\}$
Conjunction	*C*⊓*D*	$C^{{\mathcal {I}}} \cap D^{{\mathcal {I}}}$
Existential	∃*r*.*C*	$\left \{ x \in \Delta ^{{\mathcal {I}}} ~ | \exists y \in \Delta ^{{\mathcal {I}}} :\right.$
restriction		$\left.(x,y) \in r^{{\mathcal {I}}} \wedge y \in C^{{\mathcal {I}}}\right \}$
GCI	$C {\sqsubseteq } D$	$C^{{\mathcal {I}}} \subseteq D^{{\mathcal {I}}}$
RI	$r_{1} \circ \ldots \circ r_{k} {\sqsubseteq } r$	$r_{1}^{{\mathcal {I}}} \circ \ldots \circ r_{k}^{{\mathcal {I}}} \subseteq r^{{\mathcal {I}}}$

An interpretation  consists of a non-empty set $\Delta ^{{\mathcal {I}}}$ and an interpretation function $\cdot ^{{\mathcal {I}}}$ which assigns to each atomic concept *A*∈*N*_*C*_ a subset $A^{{\mathcal {I}}} \subseteq \Delta ^{{\mathcal {I}}}$, to each atomic role *r*∈*N*_*R*_ a relation $r^{{\mathcal {I}}} \subseteq \Delta ^{{\mathcal {I}}} \times \Delta ^{{\mathcal {I}}}$, and to each individual name *a*∈*N*_*I*_ an element $a^{{\mathcal {I}}} \in \Delta ^{{\mathcal {I}}}$. The interpretation function is straightforwardly extended to complex concepts. An ${\mathcal {EL}^{++}}$ TBox (named CBox in [6]) is a finite set of *general concept inclusions* (GCIs) and *role inclusions* (RIs) whose syntax can be found in the lower part of Table 1. Note that a finite set of GCIs is called a *general TBox*. An interpretation  is a *model* of a TBox *T* if for each GCI and RI in *T*, the conditions given in the third column of Table 1 are satisfied.

${\mathcal {EL}}$ has the restricted form of ${\mathcal {EL}^{++}}$ which allows for concept constructors of top concept ⊤, conjunction and existential restriction. An ${\mathcal {EL}}$ TBox contains only GCIs.

The main reasoning task for description logics is subsumption in which the problem is to decide for a TBox *T* and concepts *C* and *D* whether $T \models C {\sqsubseteq } D$. Subsumption in ${\mathcal {EL}^{++}}$ is polynomial even w.r.t. general TBoxes [6].

### Abduction framework

In the following we explain how the problem of finding possible ways to repair the missing is-a structure in a ontology is formalized as a generalized version of the TBox abduction problem as defined in [29]. We assume that our ontology is represented using a TBox *T* in a language  which in this paper is ${\mathcal {EL}}$ or ${\mathcal {EL}^{++}}$. Further, we have a set of missing is-a relations which are represented by a set *M* of atomic concept subsumptions. In *case 1* in Section Background, these missing is-a relations were detected. In *case 2* the elements in *M* are existing is-a relations in the ontology that are temporarily removed, and *T* represents the ontology that is obtained by removing the elements in *M* from the original ontology. (They can later be added again after completing the ontology.) To complete the is-a structure of an ontology, the ontology should be extended with a set *S* of atomic concept subsumptions (repair) such that the extended ontology is consistent and entails the missing is-a relations. However, the added atomic concept subsumptions should be correct according to the domain. In general, the set of all atomic concept subsumptions that are correct according to the domain are not known beforehand. Indeed, if this set were given then we would only have to add this to the ontology. The common case, however, is that we do not have this set, but instead can rely on a domain expert that can decide whether an atomic concept subsumption is correct according to the domain. In our formalization the domain expert is represented by an oracle *Or* that when given an atomic concept subsumption, returns true or false. It is then required that for every atomic concept subsumption *s*∈*S*, we have that *Or*(*s*)=*true*. The following definition formalizes this.

#### Definition 1.

(GENERALIZED TBOX ABDUCTION) Let *T* be a TBox in language  and *C* be the set of all atomic concepts in *T*. Let $M = \{ A_{i}~{\sqsubseteq }~B_{i}\}_{i = 1}^{n}$ with *A*_*i*_,*B*_*i*_∈*C* be a finite set of TBox assertions. Let $\text{\textit{Or}} : \{ C_{i} ~{\sqsubseteq }~ D_{i} \mid C_{i}, D_{i}\!\! \in \!\! C \} \rightarrow \{ true, false \}$. A solution to the generalized TBox abduction problem (GTAP) (*T*,*C*,*Or*,*M*) is any finite set of TBox assertions $S = \{ E_{i} ~{\sqsubseteq }~ F_{i}\}_{i = 1}^{k}$ such that ∀*E*_*i*_,*F*_*i*_:*E*_*i*_,*F*_*i*_∈*C*, $\forall E_{i}, F_{i}: Or(E_{i} ~{\sqsubseteq }~ F_{i}) = true$, *T*∪*S* is consistent and *T*∪*S*⊧*M*. The set of all such solutions is denoted as ${\mathcal {S}}(T, C, Or, M)$.

As an example, consider GTAP  as defined in Figure 1. Then {Carditis $\sqsubseteq $ CardioVascularDisease, InflammationProcess $\sqsubseteq $ PathologicalProcess, GranulomaProcess $\sqsubseteq $ InflammationProcess} is a solution for . Another solution is {Carditis $\sqsubseteq $ CardioVascularDisease, GranulomaProcess $\sqsubseteq $ PathologicalProcess} as shown in Section Background.

There can be many solutions for a GTAP and, as explained in Section Background, not all solutions are equally interesting. Therefore, we propose two preference criteria on the solutions as well as different ways to combine them. The first criterion is a criterion that is not used in other abduction problems, but that is particularly important for GTAP. In GTAP it is important to find solutions that add to the ontology as much information as possible that is correct according to the domain. Therefore, the first criterion prefers solutions that imply more information.

#### Definition 2.

(MORE INFORMATIVE) Let *S* and *S*^′^ be two solutions to the GTAP (*T*,*C*,*Or*,*M*). *S* is said to be *more informative* than *S*^′^ iff *T*∪*S*⊧*S*^′^ and *T*∪*S*^′^⊮*S*.

Further, we say that *S* is *equally informative* as *S*^′^ iff *T*∪*S*⊧*S*^′^ and *T*∪*S*^′^⊧*S*.

Consider two solutions to , S _1_ = {InflammationProcess $\sqsubseteq $ PathologicalProcess, GranulomaProcess $\sqsubseteq $ InflammationProcess}^d^ and *S*_2_ = {InflammationProcess $\sqsubseteq $ PathologicalProcess, GranulomaProcess $\sqsubseteq $ PathologicalProcess}. In this case solution S _1_ is more informative than S _2_.

#### Definition 3.

(SEMANTIC MAXIMALITY) A solution *S* to the GTAP (*T*,*C*,*Or*,*M*) is said to be semantically maximal iff there is no solution *S*^′^ which is more informative than *S*. The set of all semantically maximal solutions is denoted as ${\mathcal {S}}^{max}(T, C, Or, M)$.

An example of a semantically maximal solution to  is {InflammationProcess $\sqsubseteq $ PathologicalProcess, GranulomaProcess $\sqsubseteq $ InflammationProcess, Carditis $\sqsubseteq $ CardioVascularDisease}.

The second criterion is a classical criterion in abduction problems. It requires that no element in a solution is redundant.

#### Definition 4.

(SUBSET MINIMALITY) A solution *S* to the GTAP (*T*,*C*,*Or*,*M*) is said to be subset minimal iff there is no proper subset $S^{\prime } \subsetneq S$ such that *S*^′^ is a solution. The set of all subset minimal solutions is denoted as ${\mathcal {S}}_{\textit {min}}(T, C, Or, M)$.

An example of a subset minimal solution for  is {InflammationProcess $\sqsubseteq $ PathologicalProcess, GranulomaProcess $\sqsubseteq $ InflammationProcess}. On the other hand, solution {Carditis $\sqsubseteq $ CardioVascularDisease, InflammationProcess $\sqsubseteq $ PathologicalProcess, GranulomaProcess $\sqsubseteq $ InflammationProcess} is not subset minimal as it contains Carditis $\sqsubseteq $ CardioVascularDisease which is redundant for repairing the missing is-a relations.

In practice, both of the above two criteria are desirable. We therefore define ways to combine these criteria depending on what kind of priority we assign for the single preferences.

#### Definition 5.

(COMBINING WITH PRIORITY FOR SEMANTIC MAXIMALITY) A solution *S* to the GTAP (*T*,*C*,*Or*,*M*) is said to be maxmin optimal iff *S* is semantically maximal and there does not exist another semantically maximal solution *S*^′^ such that *S*^′^ is a proper subset of *S*. The set of all maxmin optimal solutions is denoted as ${\mathcal {S}}_{\textit {min}}^{\mathbf {max}}(T, C, Or, M)$.

As an example, {InflammationProcess $\sqsubseteq $ PathologicalProcess, GranulomaProcess $\sqsubseteq $ InflammationProcess, Carditis $\sqsubseteq $ CardioVascularDisease} is a maxmin optimal solution for . The advantage of maxmin optimal solutions is that a maximal body of correct information is added to the ontology and without redundancy. For GTAP these are the most attractive solutions, but it is not clear how to *generate* such solutions, except for a brute-force method^e^ that would query the oracle with, for larger ontologies, unfeasibly many questions.

#### Definition 6.

(COMBINING WITH PRIORITY FOR SUBSET MINIMALITY) A solution *S* to the GTAP (*T*,*C*,*Or*,*M*) is said to be minmax optimal iff *S* is subset minimal and there does not exist another subset minimal solution *S*^′^ such that *S*^′^ is more informative than *S*. The set of all minmax optimal solutions is denoted as ${\mathcal {S}}_{\textbf {min}}^{max}(T, C, Or, M)$.

As an example, {InflammationProcess $\sqsubseteq $ PathologicalProcess, GranulomaProcess $\sqsubseteq $ InflammationProcess} is a minmax optimal solution for . In practice, minmax optimal solutions ensure fewer is-a relations to be added, thus avoiding redundancy. This is desirable if the domain expert would prefer to look at as small solutions as possible. The disadvantage is that there may be correct relations that are not derivable when they are not included in the solution.

For the skyline interpretation, we consider the subset minimality and the semantic maximality as two dimensions for a solution *S* (see [30] for an explanation of how the definition satisfies the skyline interpretation).

#### Definition 7.

(SKYLINE OPTIMAL) A solution *S* to the GTAP (*T*,*C*,*Or*,*M*) is said to be skyline optimal iff there does not exist another solution *S*^′^ such that *S*^′^ is a proper subset of *S* and *S*^′^ is equally informative as *S*. The set of all skyline optimal solutions is denoted as ${\mathcal {S}}_{\textit {min}}^{max}(T, C, Or, M)$.

All subset minimal, minmax optimal and maxmin optimal solutions are also skyline optimal solutions. However, there are semantically maximal solutions that are not skyline optimal. For example, {InflammationProcess $\sqsubseteq $ PathologicalProcess, GranulomaProcess $\sqsubseteq $ InflammationProcess, Carditis $\sqsubseteq $ CardioVascularDisease, Endocarditis $\sqsubseteq $ CardioVascularDisease} is a semantically maximal solution for , but it is not skyline optimal as its subset {InflammationProcess $\sqsubseteq $ PathologicalProcess, GranulomaProcess $\sqsubseteq $ InflammationProcess, Carditis $\sqsubseteq $ CardioVascularDisease} is equally informative. There also exist skyline optimal solutions that are not subset minimal solutions. For instance, {InflammationProcess $\sqsubseteq $ PathologicalProcess, GranulomaProcess $\sqsubseteq $ InflammationProcess, Carditis $\sqsubseteq $ CardioVascularDisease} is a skyline optimal solution that is not subset minimal as removing Carditis $\sqsubseteq $ CardioVascularDisease would still yield a solution (although not as informative). Skyline optimal is a relaxed criterion. It requires subset minimality for some level of informativeness.

Although maxmin or semantically maximal solutions are preferred, in practice, as mentioned before, it is not clear how to *generate* such solutions, except for a brute-force method that would query the oracle with, for larger ontologies, unfeasibly many questions. Therefore, a skyline solution is the next best thing and, in the case solutions exist, it is easy to generate *a* skyline optimal solution. However, the difficulty lies in reaching an as high level of informativeness as possible.

### Complexity results

In addition to finding solutions, traditionally, there are three main decision problems for logic-based abduction: existence, relevance and necessity.

#### Definition 8.

Given a GTAP (*T*,*C*,*Or*,*M*) we define the following decision problems:

ᅟ ***Existence***${\mathcal {S}}(T, C, Or, M) \neq \emptyset $?
ᅟ ***Relevance*** Given *ψ*, does a solution $S \in {\mathcal {S}}(T, C, Or, M)$ exist such that *ψ*∈*S*?
ᅟ ***Necessity*** Given *ψ*, do all the solutions in ${\mathcal {S}}(T, C, Or, M)$ contain *ψ*?

If we replace  in Definition 8 with ${\mathcal {S}}_{\textit {min}}$, ${\mathcal {S}}^{max}$, ${\mathcal {S}}_{\textbf {min}}^{max} {\mathcal {S}}_{\textit {min}}^{\textbf {max}}$ and ${\mathcal {S}}_{\textit {min}}^{max}$, respectively, we obtain the GTAP decision problems under the criteria of subset minimality, semantic maximality and the combinations.

We have proven complexity results for these GTAP decision problems and show the summary of the results in Tables 2 (${\mathcal {EL}}$) and 3 (${\mathcal {EL}^{++}}$). For the proofs we refer to the Appendix.

**Table 2:** **Complexity results of GTAP for** ${\boldsymbol{\mathcal {EL}}}$

**Decision problems**	**Existence**	**Relevance**	**Necessity**
General	in P	in P	in P
Subset minimality	in P	NP-complete	in P
Semantic maximality	in P	in P	in P
Minmax	in P	NP-complete	in P
Maxmin	in P	in P	in P
Skyline	in P	NP-complete	in P

**Table 3:** **Complexity results of GTAP for** ${\boldsymbol{\mathcal {EL}}^{++}}$

**Decision problems**	**Existence**	**Relevance**	**Necessity**
General	NP-complete	NP-complete	co-NP-complete
Subset minimality	NP-complete	NP-complete	co-NP-complete
Semantic maximality	NP-complete	NP-complete	co-NP-complete
Minmax	NP-complete	${\Sigma _{2}^{P}}$-complete	${\Pi _{2}^{P}}$-complete
Maxmin	NP-complete	NP-complete	co-NP-complete
Skyline	NP-complete	NP-complete	co-NP-complete

While it is not surprising that with either of the single preferences of subset minimality and semantic maximality, the complexity for ${\mathcal {EL}^{++}}$ remains the same as the case without any preference, it is interesting to observe that combining the two preferences yields different complexity results. The combinations maxmin and skyline do not increase the complexity, while for minmax the complexity is higher which is at the second level of polynomial hierarchy. The intuition behind that can be explained informally as follows: for maxmin and skyline, the checking of both preference criteria can be conducted sequentially, while for minmax it is not possible. The complexity results provide a guideline on the choosing of suitable preference criteria for designing repairing algorithms in practice. As a result, the remaining part of the paper is dedicated to a concrete algorithm for finding *one* skyline optimal solution, together with a system based on the algorithm as well as experiments.

### Algorithms

In this section we present algorithms for completing the is-a structure (solving GTAP (*T*,*C*,*Or*,*M*)) in light-weight ontologies. Based on lessons learned in [30], we require that the missing is-a relations are validated before the repairing and thus ∀*m*∈*M*:*Or*(*m*)=*true*. We also require that *T*∪*M* is consistent. For ontologies represented in ${\mathcal {EL}}$ this is trivially true as all TBoxes are consistent. For ${\mathcal {EL}^{++}}$ this is a requirement for the existence of a solution to GTAP. Given these assumptions we also know that *M* is a solution.

In general, we would like to find a solution for GTAP at the highest level of informativeness. However, this can only be *guaranteed* if we know *all* missing is-a relations. As discussed before, a way to obtain this is using a brute-force method and ask *Or* for every pair in *C*×*C* whether it is a correct is-a relation according to the domain or not. In practice, for large ontologies this is not feasible. Therefore, the algorithms in this section compute initially a skyline optimal solution for GTAP (*T*,*C*,*Or*,*M*) and iteratively try to find other skyline optimal solutions at higher levels of informativeness.

As *M* is a solution, the algorithm will always return a result. The result can be a subset minimal solution that is a subset of *M* or a solution that is more informative than *M*.

In algorithm 1 we show the common part for the algorithms for the different representation languages. The algorithms contain 3 basic steps: finding a skyline-optimal solution for one missing is-a relation, finding a skyline-optimal solution for a set of missing is-a relations and finding a more informative skyline-optimal solution.



In *RepairSingleIsa* a skyline-optimal solution is found for a single missing is-a relation. This part of the algorithm is different for different knowledge representation languages and is discussed for ${\mathcal {EL}}$ and ${\mathcal {EL}^{++}}$ in Sections [Sec Sec7] and [Sec Sec8], respectively.

In *RepairMultipleIsa* the algorithm collects for each missing is-a relation a solution from *RepairSingleIsa* and takes the union of these. Therefore, the following holds for Solution in line 6: *T*∪*Solution*⊧*M* and ∀*s*∈*Solution*:*Or*(*s*)=*true*. The statements in lines 7-8 (which are redundant for ${\mathcal {EL}}$) guarantee consistency. This leads to the fact that Solution is a solution of GTAP (*T*,*C*,*Or*,*M*). Further, in line 9, we remove redundancy while keeping the same level of informativeness, and thus obtain a skyline optimal solution. (In the case where there are several ways to remove redundancy, one is chosen, as the extended ontologies will be equivalent in the sense that they entail the same statements.)

In *Repair* we try to improve the result from *RepairMultipleIsa* by trying to find a skyline optimal solution on a higher level of informativeness. Given that any element in the solution of *RepairMultipleIsa* that is not in *M* can be considered as a new missing is-a relation (which was not detected earlier), we can try to find additional more informative ways of repairing by solving a new GTAP problem for these new missing is-a relations (and continue as long as new missing is-a relations are detected). As a (skyline optimal) solution for the new GTAP is also a (skyline optimal) solution of the original GTAP, the solution found in *Repair* is a skyline optimal solution for the original GTAP.

### Algorithm - ${\mathcal {EL}}$

We now present an algorithm for *RepairSingleIsa* for ontologies that are represented in ${\mathcal {EL}}$ and where the TBox is normalized as described in [6]. A normalized TBox *T* contains only axioms of the forms $A_{1} \sqcap \dots \sqcap A_{n} \sqsubseteq B$, $A \sqsubseteq \exists r.B$, and $\exists r.A \sqsubseteq B$, where *A*, *A*_1_, …, *A*_*n*_ and *B* are atomic concepts and *r* is a role. Every ${\mathcal {EL}}$ TBox can in linear time be transformed into a normalized TBox that is a conservative extension, i.e., every model of the normalized TBox is also a model of the original TBox and every model of the original TBox can be extended to a model of the normalized TBox.

The algorithm in Algorithm 2 computes a solution for a GTAP with one missing is-a relation (i.e. GTAP $(T, C, Or, \{E \sqsubseteq F\})$ in the following way. First, superconcepts of E are collected in a *Source* set and subconcepts of F are collected in a *Target* set (lines 3 and 4). *Source* contains expressions of the forms *A* and ∃*r*.*A* while *Target* contains expressions of the forms *A*, *A*_1_⊓⋯⊓*A*_*n*_ and ∃*r*.*A* where *A*, *A*_1_, …, *A*_*n*_ are atomic concepts and *r* is a role. Adding an is-a relation between an element in Source and an element in Target to the ontology would make $E~ \sqsubseteq ~ F$ derivable (and thus this gives us logical solutions, but not necessarily solutions that are correct according to the domain). As we are interested in solutions containing is-a relations between atomic concepts, we check for every pair (A,B) ∈ Source × Target whether A and B are atomic concepts and *Or*($A ~\sqsubseteq ~B$) = *true* (i.e. correct according to the domain). If so, then this is a possible solution for GTAP $(T, C, Or, \{E ~\sqsubseteq ~ F\})$. However, to conform to subset minimality and semantic maximality, if the current solution already contains is-a relations that would lead to the entailment of $A~ \sqsubseteq ~ B$ then we do not use $A~ \sqsubseteq ~ B$ (8-9). Otherwise we use $A~ \sqsubseteq ~ B$ and remove elements from the current solution that would be entailed if $A~ \sqsubseteq ~ B$ is used (10-12). Further, in the case where A is of the form ∃*r*.*N* and B is of the form ∃*r*.*O*, then making $N~ \sqsubseteq ~ O$ derivable would also make $A ~\sqsubseteq ~ B$ derivable (14-15)^f^. It is clear that for the result of *RepairSingleIsa*, i.e. Sol, the following holds: $T \cup Sol \models E~ \sqsubseteq ~ F$ and ∀*s*∈*Sol*:*Or*(*s*)=*true*. Together with the fact that $\mathcal {EL}$ TBoxes are consistent, this leads to the fact that Sol is a solution of GTAP $(T, C, Or, \{E ~\sqsubseteq ~ F\})$.



As an example run for the solving GTAP for ${\mathcal {EL}}$ ontologies, consider the GTAP in Figure 1. For a given ontology and set of missing is-a relations, the algorithm will first find solutions for repairing individual missing is-a relations using *RepairSingleIsA*. For the missing is-a relation Endocarditis $\sqsubseteq $ PathologicalPhenomenon the following is-a relations, when added to the ontology, would allow to derive the missing is-a relation: Endocarditis $\sqsubseteq $ PathologicalPhenomenon, Endocarditis $\sqsubseteq $ Fracture, Endocarditis $\sqsubseteq $ CardioVascularDisease, Carditis $\sqsubseteq $ PathologicalPhenomenon, Carditis $\sqsubseteq $ Fracture, Carditis $\sqsubseteq $ CardioVascularDisease as well as InflammationProcess $\sqsubseteq $ PathologicalProcess. As the first one is the missing is-a relation which was already validated, only the other six is-a relations are presented to the oracle for validation. Out of these six Endocarditis $\sqsubseteq $ Fracture and Carditis $\sqsubseteq $ Fracture are not correct according to the domain and are therefore not included in solutions. Further, relations Endocarditis $\sqsubseteq $ CardioVascularDisease, Endocarditis $\sqsubseteq $ PathologicalPhenomenon, Carditis $\sqsubseteq $ PathologicalPhenomenon are removed given it is possible to entail them from the ontology together with the remaining relations. Therefore, after validation, *RepairSingleIsA* returns {InflammationProcess $\sqsubseteq $ PathologicalProcess, Carditis $\sqsubseteq $ CardioVascularDisease}. The same process is repeated for the second missing is-a relation GranulomaProcess $\sqsubseteq $ NonNormalProcess. In this case the following is-a relations, when added to the ontology, would allow to derive the missing is-a relation: GranulomaProcess $\sqsubseteq $ NonNormalProcess and GranulomaProcess $\sqsubseteq $ PathologicalProcess. GranulomaProcess $\sqsubseteq $ NonNormalProcess is the missing is-a relation and was already validated as correct according to the domain. GranulomaProcess $\sqsubseteq $ PathologicalProcess is presented to the oracle and validated as correct according to the domain. As GranulomaProcess $\sqsubseteq $ NonNormalProcess can be entailed from the ontology together with GranulomaProcess $\sqsubseteq $ PathologicalProcess, *RepairSingleIsA* returns {GranulomaProcess $\sqsubseteq $ PathologicalProcess}. The solutions for the single is-a relations are then combined to form a solution for the set of missing is-a relations. In our case, there are no redundant relations and therefore *RepairMultipleIsA* returns {InflammationProcess $\sqsubseteq $ PathologicalProcess, Carditis $\sqsubseteq $ CardioVascularDisease, GranulomaProcess $\sqsubseteq $ PathologicalProcess}. We note that this is a skyline optimal solution. In *Repair* the system tries to improve the acquired solution. This time the oracle is presented with a total of 13 relations for validation out of which only one is validated to be correct, i.e. GranulomaProcess $\sqsubseteq $ InflammationProcess. This is added to the solution. Given this new is-a relation, GranulomaProcess $\sqsubseteq $ PathologicalProces is removed from the solution as it can now be entailed from the ontology and GranulomaProcess $\sqsubseteq $ InflammationProcess. The new solution is {InflammationProcess $\sqsubseteq $ PathologicalProcess, Carditis $\sqsubseteq $ CardioVascularDisease, GranulomaProcess $\sqsubseteq $ InflammationProcess}. This is again a skyline optimal solution and it is more informative than the previous solution. As new missing is-a relations were detected, the repairing is run for the third time. However, in this run the solution is not improved and thus the algorithm outputs the final result. We note that in this example we found a skyline optimal solution that is also semantically maximal. In general, however, it is not possible to know whether the solution is semantically maximal without checking every possible is-a relation between atomic concepts in the ontology.



#### Algorithm - ${\mathcal {EL}^{++}}$

We now present an algorithm for *RepairSingleIsa* for ontologies that are represented in ${\mathcal {EL}^{++}}$ (Algorithm 3) and where the TBox is normalized as described in [6]. A normalized TBox *T* contains only axioms of the forms $A_{1} \sqcap \dots \sqcap A_{n} \sqsubseteq B$, $A \sqsubseteq \exists r.B$, and $\exists r.A \sqsubseteq B$, as well as role inclusions of the forms $r \sqsubseteq s$ and $r_{1} \circ r_{2} \sqsubseteq s$ where *A*, *A*_1_, …, *A*_*n*_ and *B* are atomic concepts and *r*, *r*_1_, *r*_2_ and *s* are roles. We note that, as for ${\mathcal {EL}}$ TBoxes, every ${\mathcal {EL}^{++}}$ TBox can in linear time be transformed into a normalized TBox that is a conservative extension of the original TBox.

The main difference with respect to the algorithm for ${\mathcal {EL}}$ ontologies is that the algorithm for ${\mathcal {EL}^{++}}$ needs to take into account role inclusions when searching for solutions which are found using axioms containing ∃ expressions. This is shown in lines 15-19 and *FindExistsSolutions*. As in the algorithm for ${\mathcal {EL}}$, if A is of the form ∃*r*.*N* and B is of the form ∃*r*.*O*, then making $N~ \sqsubseteq ~ O$ derivable would also make $A ~\sqsubseteq ~ B$ derivable. In ${\mathcal {EL}^{++}}$ there are two more possibilities when A is of the form ∃*r*.*N* and B is of the form ∃*s*.*O*. If *T* contains $r\sqsubseteq s$, then making $N~ \sqsubseteq ~ O$ derivable would also make $A ~\sqsubseteq ~ B$ derivable. Further, if *T* contains $r \circ r_{1} \sqsubseteq s$ and $N \sqsubseteq \exists r_{1}.P$, then making $P~ \sqsubseteq ~ O$ derivable would also make $A ~\sqsubseteq ~ B$ derivable.

**Figure 3:**
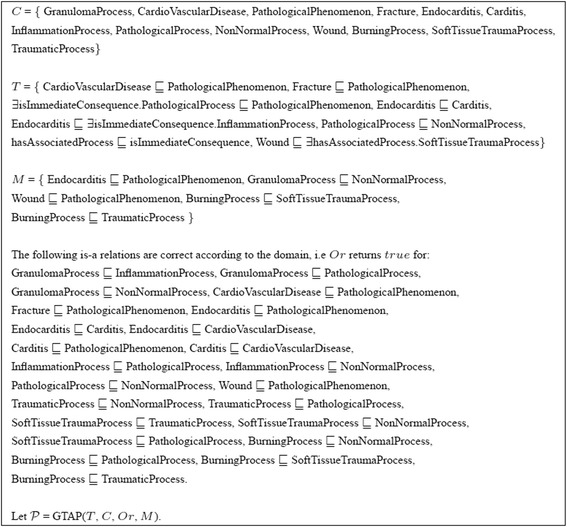
Small ${\mathcal {EL}^{++}}$ example. (C is the set of atomic concepts in the ontology. T is a TBox representing the ontology. M is a set of missing is-a relations. Or is the oracle representing the domain expert).

**Figure 4:**
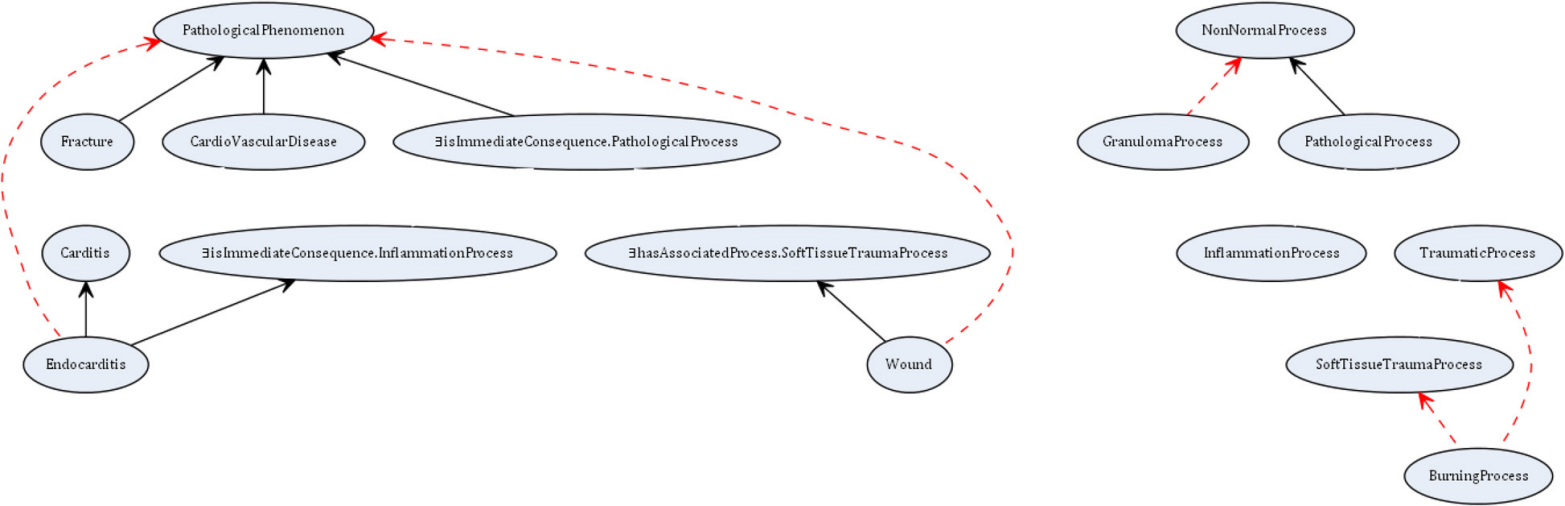
Graphical representation of the ${\mathcal {EL}^{++}}$ example in Figure 3. (Ovals represent concepts. Full arrows represent is-a relations between concepts in the ontology. Dashed arrows represent missing is-a relations).

As an example run for the solving GTAP for ${\mathcal {EL}^{++}}$ ontologies, consider the GTAP in Figure 3 (and Figure 4). For a given ontology and set of missing is-a relations, the algorithm will first find solutions for repairing individual missing is-a relations using *RepairSingleIsA*. For the missing is-a relation Endocarditis $\sqsubseteq $ PathologicalPhenomenon the following is-a relations, when added to the ontology, would allow to derive the missing is-a relation: Endocarditis $\sqsubseteq $ PathologicalPhenomenon, Endocarditis $\sqsubseteq $ Fracture, Endocarditis $\sqsubseteq $ CardioVascularDisease, Carditis $\sqsubseteq $ PathologicalPhenomenon, Carditis $\sqsubseteq $ Fracture, Carditis $\sqsubseteq $ CardioVascularDisease as well as InflammationProcess $\sqsubseteq $ PathologicalProcess. As the first one is the missing is-a relation which was already validated, only the other six is-a relations are presented to the oracle for validation. Out of these six Endocarditis $\sqsubseteq $ Fracture and Carditis $\sqsubseteq $ Fracture are not correct according to the domain and are therefore not included in solutions. Further, relations Endocarditis $\sqsubseteq $ CardioVascularDisease, Endocarditis $\sqsubseteq $ PathologicalPhenomenon, Carditis $\sqsubseteq $ PathologicalPhenomenon are removed given it is possible to entail them from the ontology together with the remaining relations. Therefore, after validation, *RepairSingleIsA* returns {InflammationProcess $\sqsubseteq $ PathologicalProcess, Carditis $\sqsubseteq $ CardioVascularDisease}. The same process is repeated for the second missing is-a relation GranulomaProcess $\sqsubseteq $ NonNormalProcess. In this case the following is-a relations, when added to the ontology, would allow to derive the missing is-a relation: GranulomaProcess $\sqsubseteq $ NonNormalProcess and GranulomaProcess $\sqsubseteq $ PathologicalProcess. GranulomaProcess $\sqsubseteq $ NonNormalProcess is the missing is-a relation and was already validated as correct according to the domain. GranulomaProcess $\sqsubseteq $ PathologicalProcess is presented to the oracle and validated as correct according to the domain. As GranulomaProcess $\sqsubseteq $ NonNormalProcess can be entailed from the ontology together with GranulomaProcess $\sqsubseteq $ PathologicalProcess, *RepairSingleIsA* returns {GranulomaProcess $\sqsubseteq $ PathologicalProcess}. For the missing is-a relation Wound $\sqsubseteq $ PathologicalPhenomenon relations Wound $\sqsubseteq $ PathologicalPhenomenon, SoftTissueTraumaProcess $\sqsubseteq $ PathologicalProcess, Wound $\sqsubseteq $ Fracture, Wound $\sqsubseteq $ CardioVascularDisease, when added to the ontology, would allow to derive the missing is-a relation. Out of these, only Wound $\sqsubseteq $ PathologicalPhenomenon and SoftTissueTraumaProcess $\sqsubseteq $ PathologicalProcess are correct according to the oracle, and *RepairSingleIsA* therefore returns {Wound $\sqsubseteq $ PathologicalPhenomenon, SoftTissueTraumaProcess $\sqsubseteq $ PathologicalProcess}. For the remaining missing is-a relations BurningProcess $\sqsubseteq $ SoftTissueTraumaProcess and BurningProcess $\sqsubseteq $ TraumaticProcess the procedure *RepairSingleIsA* returns {BurningProcess $\sqsubseteq $ SoftTissueTraumaProcess} and {BurningProcess $\sqsubseteq $ TraumaticProcess} respectively. The solutions for the single is-a relations are then combined to form a solution for the set of missing is-a relations. In our case, Wound $\sqsubseteq $ PathologicalPhenomenon is redundant and therefore *RepairMultipleIsA* returns {InflammationProcess $\sqsubseteq $ PathologicalProcess, Carditis $\sqsubseteq $ CardioVascularDisease, GranulomaProcess $\sqsubseteq $ PathologicalProcess, BurningProcess $\sqsubseteq $ TraumaticProcess, BurningProcess $\sqsubseteq $ SoftTissueTraumaProcess, SoftTissueTraumaProcess $\sqsubseteq $ PathologicalProcess}. We note that this is a skyline optimal solution. In *Repair* the system tries to improve the acquired solution. This time the oracle is presented with a total of 25 relations for validation out of which only two are validated to be correct, i.e. GranulomaProcess $\sqsubseteq $ InflammationProcess and SoftTissueTraumaProcess $\sqsubseteq $ TraumaticProcess. These are added to the solution. Given these new is-a relations, GranulomaProcess $\sqsubseteq $ PathologicalProcess and BurningProcess $\sqsubseteq $ TraumaticProcess are removed from the solution as they are redundant. The new solution is {InflammationProcess $\sqsubseteq $ PathologicalProcess, Carditis $\sqsubseteq $ CardioVascularDisease, GranulomaProcess $\sqsubseteq $ InflammationProcess, SoftTissueTraumaProcess $\sqsubseteq $ TraumaticProcess, BurningProcess $\sqsubseteq $ SoftTissueTraumaProcess, SoftTissueTraumaProcess $\sqsubseteq $ PathologicalProcess}. This is again a skyline optimal solution and it is more informative than the previous solution.

As new missing is-a relations were detected, the repairing is run for the third time. In this iteration 5 relations required validation and only relation TraumaticProcess $\sqsubseteq $ PathologicalProcess is validated as correct according to the domain. The new solution is {InflammationProcess $\sqsubseteq $ PathologicalProcess, Carditis $\sqsubseteq $ CardioVascularDisease, GranulomaProcess $\sqsubseteq $ InflammationProcess, SoftTissueTraumaProcess $\sqsubseteq $ TraumaticProcess, BurningProcess $\sqsubseteq $ SoftTissueTraumaProcess, TraumaticProcess $\sqsubseteq $ PathologicalProcess}. The relation SoftTissueTraumaProcess $\sqsubseteq $ PathologicalProcess was removed from the solution as it is redundant.

The algorithm is run again and in this iteration no new is-a relations were validated to be correct so the solution from the previous iteration is returned as the final solution.

### System

We have implemented a system for repairing missing is-a relations. The input to the system is an ontology in ${\mathcal {EL}}$ or ${\mathcal {EL}^{++}}$ and a set of validated missing is-a relations. The output is a solution to GTAP (called a *repairing action*). The system was implemented in Java and uses the ELK reasoner (version 0.4.1) [31] to detect implicit entailments in the ontology. The system is semi-automatic and requires interaction with a user which is a domain expert^g^ serving as an oracle and who decides whether an is-a relation is correct according to the domain.

**Figure 5:**
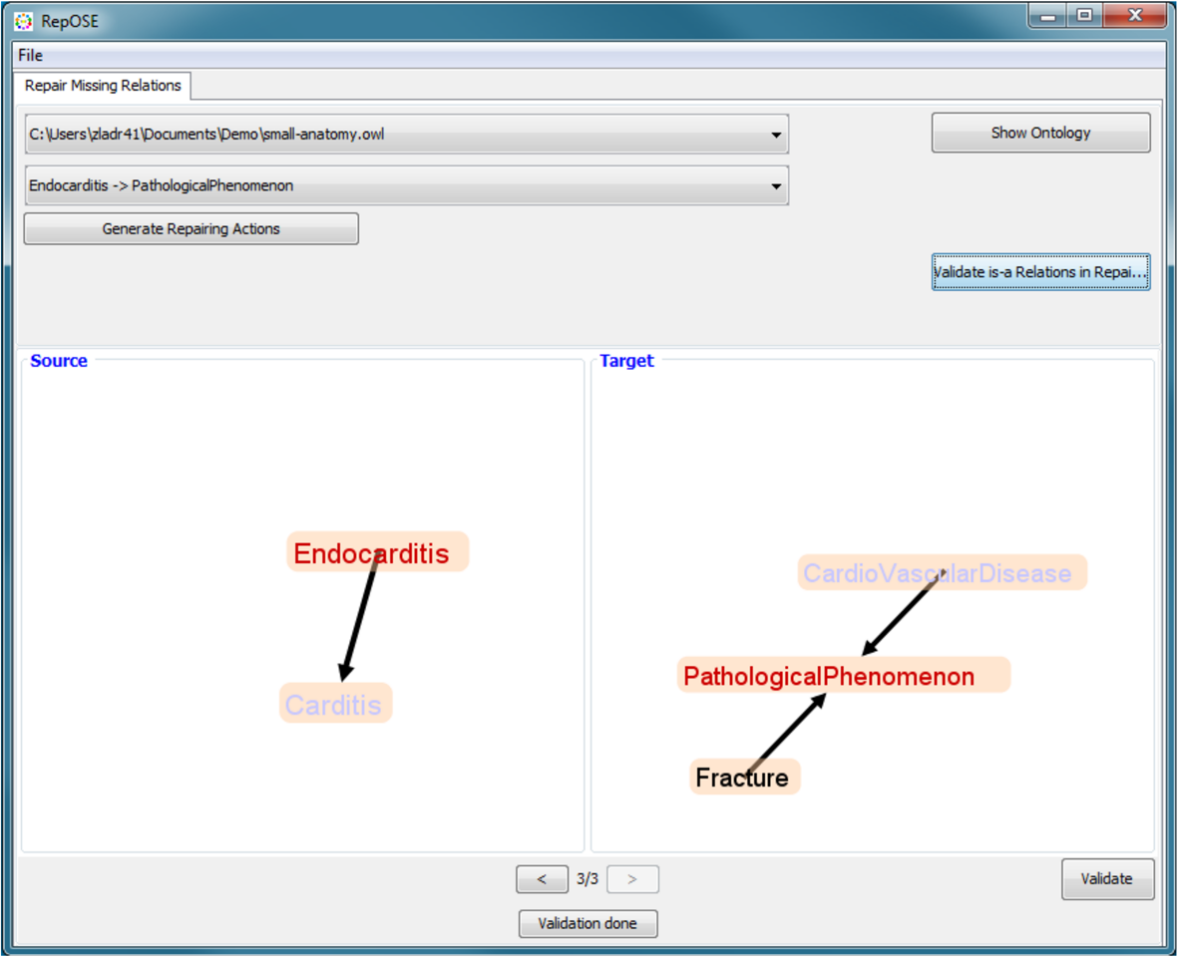
Screenshot - repairing using source and target sets.

Once the ontology and the set of missing is-a relations are loaded, the user starts the debugging process by pressing the button Generate Repairing Actions (Figure 5). The system then removes redundant is-a relations and the non-redundant missing is-a relations are shown in a drop-down list allowing the user to switch between missing is-a relations. Additional relations acquired using ∃ expressions are also included in the drop-down list. It is also possible to scroll between relations using the arrow buttons in the bottom part of the screen.

After selecting an is-a relation from the list, the user is presented with the Source and the Target set for that is-a relation. The user then needs to choose relations which are correct according to the domain for that is-a relation. Missing is-a relations are automatically validated to be correct according to the domain while the relations that were acquired using ∃ expressions have to be explicitly validated by the user.

In Figure 5 the user is presented with the Source and the Target set for the missing is-a relation Endocarditis $\sqsubseteq $ PathologicalPhenomenon (concepts in the missing is-a relation are marked in red). In this case the user has selected {Carditis $\sqsubseteq $ CardioVascularDisease} as a repairing action for the missing is-a relation (concepts marked in purple) and needs to confirm this by clicking the Validate button.

**Figure 6:**
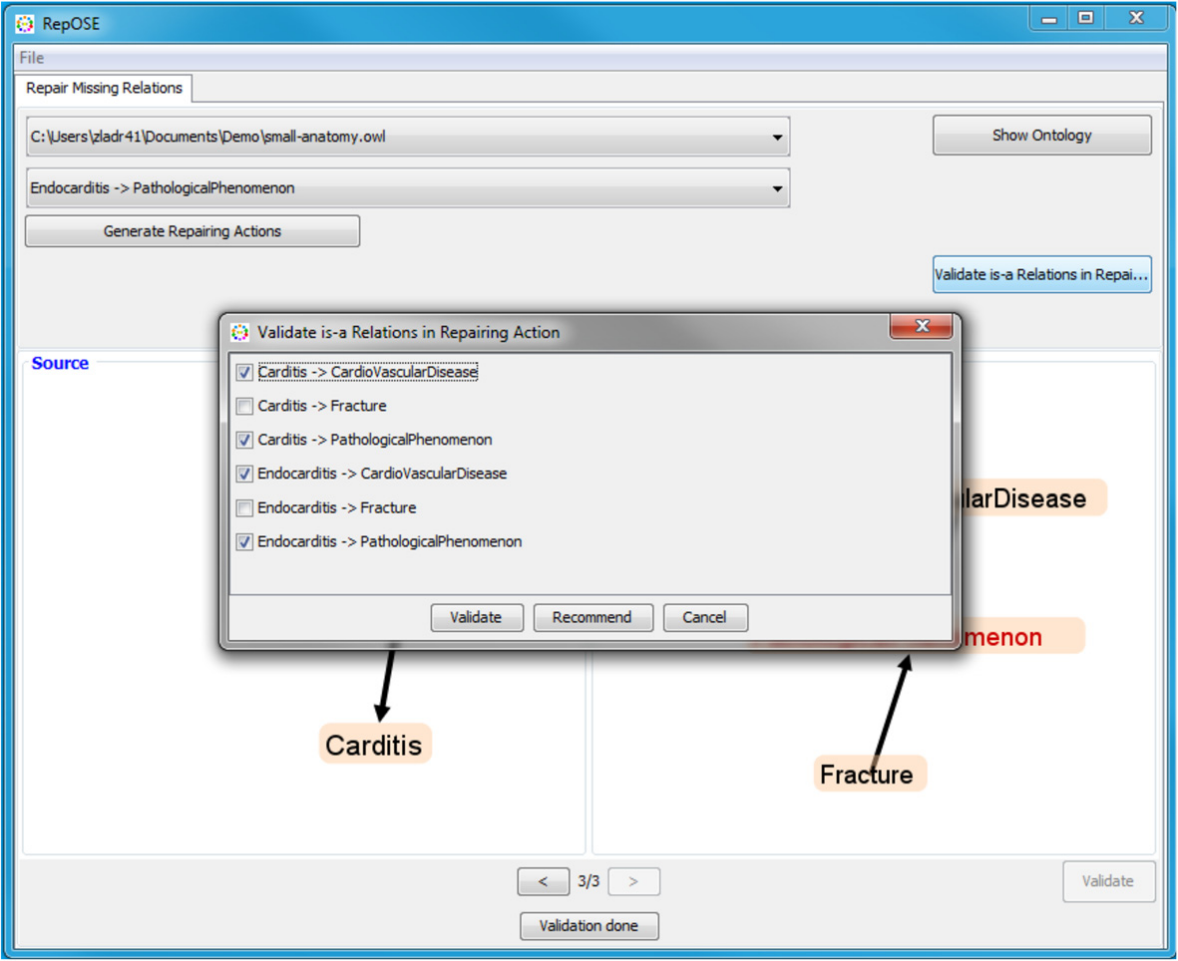
Screenshot - validating is-a relations in a repairing action.

The user also has the option to check which relations have been validated so far and which relations can be validated, by clicking the Validate Is-a Relations button. In the pop-up window that appears the user can validate new relations, remove validations from already validated relations as well as ask for a recommendation by clicking the Recommend button (Figure 6). Recommendations are acquired by querying external sources (currently, WordNet [32], UMLS Methathesaurus and Uberon [33]) by checking for the pairs consisting of a concept in Source and a concept in Target whether there is an is-a relation between these in the external source^h^.

The validation phase is ended by clicking on the Validation Done button. The system then calculates the consequences of the chosen repairing actions and presents the user with a new set of is-a relations that need to be repaired. The validation phase and consequent computations represent one iteration of the Repair procedure in Algorithm 2. If the repairing did not change between two iterations the system outputs the repairing.

At any point the user can save validated relations from the "File" menu which makes it possible to do debugging accross multiple sessions.

## Experiments

We have run several debugging experiments. Our goal was to investigate the usefulness of our approach in cases 1 and 2 and for real ontologies. Therefore, we developed experiments for cases 1 and 2 and used existing ontologies regarding anatomy (case 1) and Biotop (case 2). The question about usefulness was divided into two parts. First, we wanted an indication of the additional knowledge that was added to the ontology. For this we measure the number of newly found is-a relations. Further, we wanted an indication of the required user interaction with the domain expert who needs to validate the solutions. For this we measure the number of and sizes of Source and Target sets which represent all the logical solutions found by our system.

The experiments were performed on an Intel Core i7-2620M Processor at 3.07 GHz with 4 GB RAM under Windows 7 Professional and Java 1.7 compiler. In all experiments the validation phase took the most time while the computations between iterations took less than 10 seconds.

The results are summarized in Tables 4, 5, 6, 7 and 8. The ’It’ columns represent the different iterations of Repair in Algoritm 1. The ’Missing’ rows give the number of missing is-a relations in each iteration. For instance, in Table 5 in the first iteration, there are the 5 original missing is-a relations. Such a missing is-a relation can be repaired by adding itself (’Repaired by itself’), or by adding other is-a relations that were not derivable in the ontology extended with the missing is-a relations and thus represent new knowledge added to the ontology (’Repaired using new knowledge’). The ’New relations’ row shows how many new is-a relations were added to the ontology to repair the missing is-a relations which were repaired using new knowledge. When such relations were found using ∃ (e.g., lines 14-15 in Algorithm 2 or lines 15-19 in Algorithm 3), then the number of such relations is shown in parentheses. For instance, in Table 5, in the first iteration 3 original missing is-a relations were repaired by adding 4 new relations representing new knowledge of which 2 were found using ∃. We note that for iteration *i*+1 the missing is-a relations (row ’Missing’) are obtained by taking the union of the missing is-a relations repaired by themselves from iteration *i* and the new relations from iteration *i* that were used to repair the other missing is-a relations in iteration *i*, and then removing the redundant relations from this set. For instance, in Table 5, for the second iteration the missing is-a relations are the 2 original is-a relations that were repaired by adding themselves and the 4 new is-a relations that were added for repairing the 3 other original missing is-a relations. As there are no redundant relations among these, the number of missing is-a relations in iteration 2 is 6. We also note that in the *last* iteration all missing is-a relations from that iteration are always repaired by themselves and these represent the final repairing action.

**Table 4:** **Results for the small ontology in Figure** 1

	**It1**	**It2**	**It3**
Missing	2	3	3
Repaired by itself	0	2	3
Repaired using new knowledge	2	1	0
New relations	3(1)	1	0

**Table 5:** **Results for the small ontology in Figure** 3

	**It1**	**It2**	**It3**
Missing	5	6	6
Repaired by itself	2	4	6
Repaired using new knowledge	3	2	0
New relations	4(2)	2	0

**Table 6:** **Results for debugging AMA - Mouse Anatomy ontology**

	**It1**	**It2**	**It3**
Missing	94	101	101
Repaired by itself	57	98	101
Repaired using new knowledge	37	3	0
New relations	44	3	0

**Table 7:** **Results for debugging NCI-A - Human Anatomy ontology**

	**It1**	**It2**	**It3**
Missing	58	55	54
Repaired by itself	49	50	54
Repaired using new knowledge	9	5	0
New relations	6	4	0

**Table 8:** **Results for debugging the Biotop ontology**

	**It1**	**It2**	**It3**	**It4**
Missing	47	41	42	41
Repaired by itself	19	31	38	41
Repaired using new knowledge	28	10	4	0
New relations	26(3)	11	3(1)	0

For the example in Figure 1 the system behaves as explained in Section [Sec Sec7] and the results are summarized in Table 4. The results for the example in Figure 3 are given in Table 5. Further, we performed experiments for the two different cases (missing is-a relations given or not) with existing biomedical ontologies.

During a session the user is presented with Source and Target sets for each of the current missing is-a relations. To add an is-a relation to the ontology the user chooses an element from the Source set and an element from the Target set. Multiple such is-a relations may be chosen for each shown pair of Source and Target set. In Tables 9, 10 and 11 we show the number of Source and Target sets of particular sizes for the different iterations of the algorithm. For instance, Table 9 shows that there were three iterations of the algoritm (cells have 3 values x/y/z). In the first iteration (‘x’ values), there were 56 Source sets of size 1 and 38 of size between 2 and 10, while there were 34 Target sets of size 1, 12 of size between 2 and 10, 10 of size between 11 and 20, 3 of size between 31 and 40, 6 of size between 41 and 50, 4 of size between 51 and 100, 21 of size between 101 and 200, and 4 of size between 301 and 400. The numbers for the second and third iteration are represented by the ‘y’ and ‘z’ values, respectively.

**Table 9:** **Source and target set sizes for debugging AMA - Mouse Anatomy ontology**

	**1**	**2-10**	**11-20**	**21-30**	**31-40**
AMA - Source	56/66/67	38/35/34	0/0/0	0/0/0	0/0/0
AMA - Target	34/12/12	12/43/43	10/20/21	0/1/1	3/3/3
	41-50	51-100	101-200	201-300	301-400
AMA - Source	0/0/0	0/0/0	0/0/0	0/0/0	0/0/0
AMA - Target	6/6/6	4/3/3	21/12/11	0/1/1	4/0/0

(The x/y/z values represent the sizes for iteration 1, 2 and 3, respectively).

**Table 10:** **Source and target set sizes for debugging NCI-A - Human Anatomy ontology**

	**1**	**2-10**	**11-20**	**21-30**	**31-40**
NCI-A - Source	17/23/22	41/32/32	0/0/0	0/0/0	0/0/0
NCI-A - Target	35/7/9	12/35/32	3/5/5	0/1/1	0/0/0
	41-50	51-100	101-200	201-300	301-400
NCI-A - Source	0/0/0	0/0/0	0/0/0	0/0/0	0/0/0
NCI-A - Target	0/0/0	4/3/3	1/2/2	1/1/1	2/1/1

(The x/y/z values represent the sizes for iteration 1, 2 and 3, respectively).

**Table 11:** **Source and target set sizes for debugging the Biotop ontology**

	**1**	**2-10**	**11-20**	**21-30**	**31-40**
BioTop - Source	26/44/48/53	24/18/15/0	0/0/0/0	0/0/0/0	0/0/0/0
BioTop - Target	9/15/17/13	28/22/23/19	5/6/6/6	1/10/8/7	0/2/2/2
	41-50	51-100	101-200	201-300	301-400
BioTop - Source	0/0/0/0	0/0/0/0	0/0/0/0	0/0/0/0	0/0/0/0
BioTop - Target	0/1/2/0	7/1/2/4	0/5/2/1	0/0/1/1	0/0/0/0

(The x/y/z/u values represent the sizes for iteration 1, 2, 3 and 4, respectively).

### Case 1 experiment - OAEI anatomy

We debugged the two ontologies from the Anatomy track at the 2013 Ontology Alignment Evaluation Initiative, i.e. Mouse Anatomy ontology (AMA) containing 2744 concepts and 4493 asserted is-a relations and a fragment of NCI human anatomy ontology (NCI-A) containing 3304 concepts and 5423 asserted is-a relations. The input missing is-a relations for these two experiments were a set of 94 and 58 missing is-a relations, respectively, for AMA and NCI-A. These missing is-a relations were obtained by using a logic-based approach using an alignment between AMA and NCI-A [34] to generate candidate missing is-a relations which were then validated by a domain expert to obtain actual missing is-a relations. Therefore, this experiment is related to *case 1*. We note that due to the lack of axioms involving ∃ in these ontologies, no solutions are found using ∃ (i.e., there are no numbers in parentheses in the ’New relations’ rows).

#### Mouse anatomy

The results for debugging AMA are given in Table 6. Three iterations were required to reach the final solution. Out of 94 initial missing is-a relations 37 were repaired by repairing actions which add new knowledge to the ontology while 57 were repaired using only the missing is-a relation itself. There were no derivable relations. In total 44 new and non-redundant relations were added to the ontology in the first iteration. Out of 37 relations which were repaired by adding new relations, 22 had more than 1 non-redundant relation in the repairing action. For example, the missing is-a relation wrist joint $\sqsubseteq $ joint is repaired by a repairing action {limb joint $\sqsubseteq $ joint, wrist joint $\sqsubseteq $ synovial joint}.

The set of missing is-a relations in the second iteration contains 101 relations, i.e. 57 relations which were repaired by adding the missing is-a relation itself and 44 newly added relations. In this iteration, 3 is-a relations were repaired by adding new knowledge to the ontology. All 3 of these is-a relations are is-a relations which were added in the previous iteration. For example, is-a relation wrist joint $\sqsubseteq $ synovial joint is repaired by a repairing action {wrist joint $\sqsubseteq $ hand joint} which is possible given that the is-a relation metacarpo-phalangeal joint $\sqsubseteq $ joint from the initial set of missing is-a relations was repaired by a repairing action {hand joint $\sqsubseteq $ synovial joint, limb joint $\sqsubseteq $ joint} in the first iteration. Finally, the set of missing is-a relations containing 101 is-a relations in the third iteration is also the solution for the initial set of missing is-a relations given that no new relations were added in the third iteration.

The sizes for the Source and Target sets for the different iterations are given in Table 9. We note that many sets have size 1 and most of the sets have size up to 10. This means that it is easy to visualize these sets in the system and the cognitive effort for the user is not so high. For some sets there are too many elements to have a suitable visualization in the current system.

#### NCI - human anatomy

The results for debugging NCI-A are given in Table 7. The initial set of missing is-a relations contained 58 relations. Out of these 58 relations in the first iteration 9 were repaired by adding relations which introduce new knowledge to the ontology. In total 6 new is-a relations were added and 4 missing is-a relations were derivable.

In the second iteration, 5 out of 55 is-a relations were repaired by adding new relations while repairing actions for the 50 other is-a relations were unchanged. All 5 is-a relations which were repaired by adding new relations to the ontology are is-a relations which were repaired by repairing actions containing only the missing is-a relation from the first iteration. This exemplifies why it is beneficial to consider already repaired is-a relations in subsequent iterations as Source and Target sets for some missing is-a relations can change and more informative solutions might be identified.

The input to the third iteration is a set of 54 is-a relations and given that no changes were made, these relations are the final solution.

The sizes for the Source and Target sets for the different iterations are given in Table 10. The same comments as for the AMA experiment hold for this experiment.

### Case 2 experiment - Biotop

This experiment relates to Case 2. In this experiment we used the Biotop ontology from the 2013 OWL Reasoner Evaluation Workshop dataset containing 280 concepts and 42 object properties as well as 267 asserted is-a relations and 65 asserted equivalence relations. For the set of missing is-a relations we randomly selected 47 is-a relations. Then the ontology was modified by removing is-a relations which would make the selected is-a relations derivable. The unmodified ontology was used as domain knowledge in the experiment. The results for debugging Biotop ontology are presented in Table 8.

The debugging process took 4 iterations. In the first iteration 28 relations were repaired by adding new relations. In total 26 new relations were added in the first iteration using axioms containing ∃ expressions. For example, for missing is-a relation GreatApe $\sqsubseteq $ Primate we have a repairing action {FamilyHominidaeQuality $\sqsubseteq $ OrderPrimatesQuality} given that the ontology contains axioms GreatApe $\sqsubseteq \exists $hasInherence.FamilyHominidaeQuality and ∃hasInherence.OrderPrimatesQuality $\sqsubseteq $ Primate.

The input to the second iteration contained 41 non-redundant is-a relations (4 redundant is-a relations were removed from the solution in iteration 1). In total 10 is-a relations were repaired by adding new is-a relations. Out of these 10 repaired is-a relations, 5 are relations from the initial set of missing is-a relations while the other 5 are relations which were added in the first iteration. For example, is-a relation Atom $\sqsubseteq $ Entity from the initial set of missing relations can be repaired with {Atom $\sqsubseteq $ MaterialEntity} given that MaterialEntity $\sqsubseteq $ Entity was added in the previous iteration.

In the third iteration, the input contained 42 is-a relations. In total 4 is-a relations (3 from the initial set of missing is-a relations and 1 from iteration 1) were repaired by adding 3 new relations. Out of the 3 new relations 1 is acquired using axioms containing ∃ expressions.

Finally, in the fourth iteration no new relations were added and the system outputs the solution.

During the repairing we found two new is-a relations that could not be derived from the original ontology and thus constitute new knowledge.

The sizes for the Source and Target sets for the different iterations are given in Table 11. Similar comments as for the AMA and NCI-A experiments hold for this experiment.

## Discussion

We have formalized the completing of missing is-a structure in ontologies as a GTAP, an abduction problem. However, there are several properties of completing the is-a structure in ontologies which distinguish themselves from the classic abduction framework. First, in the classic abduction framework there is a hypothesis *H* from which the solution *S* is chosen such that *S*⊆*H* holds. The corresponding component in the completing of is-a structure is the set of atomic concept subsumptions that should be correct according to the domain. In general, this set is not known beforehand. In the repairing scenario, a domain expert decides whether an atomic concept subsumption is correct according to the domain, and can return *true* or *false* like an oracle. Consequently, in the formalization we have an oracle *Or*, rather than a hypothesis set *H*. This has also an impact in how solutions can be found. In the classic abduction problem finding solutions can start from *H*. In GTAP this is not possible, but (partial) solutions are validated using *Or*. Secondly, in completing missing is-a structure a more *informative* solution is preferred to a less informative one where informativeness is a measurement for how much information the added subsumptions (i.e. solution *S*) can derive. This is in contrast to the criteria of minimality (e.g. subset minimality, cardinality minimality) from the classic abduction framework. In principle this difference on the preference stems from the original purpose of the two formalisms. The abduction framework is often used for diagnostic scenarios, thus the essential goal is to confine the cause of the problem as small as possible. Whilst for ontology repairing, the goal is to add more subsumptions to enrich the ontology. As long as the added rules are correct, a more informative repairing means more enrichment to the ontology.

The experiments have shown the usefulness of our approach. In each of the cases, whether missing is-a relations were identified, or whether we investigated existing is-a relations, our approach identified new information to be added to the ontologies.

The experiments have also shown that the iterative approach to repairing missing is-a relations is beneficial as in all our experiments additional relations were added to the ontology in subsequent iterations. Running the system on already repaired is-a relations gives the opportunity to identify new repairing actions which introduce new knowledge to the ontology. An example of this is found in the BioTop experiment where is-a relations from the initial set of missing is-a relations were repaired by more informative solutions in the third iteration.

High-quality debugging of modeling defects always requires validation by a domain expert and this is thus also the case for the completing of the is-a structure in ontologies. For each of the missing is-a relations a domain expert has to validate the generated solutions. In our system the solutions are shown in groups using the Source and Target sets. This allows the domain expert to (i) look at different related solutions at the same time and (ii) have a context for the solutions. For AMA the user looked at 94, 101 and 101 Source and Target sets in the three iterations, respectively. For NCI-A this was 58, 55 and 54, respectively. For these 2 ontologies the number of Source-Target sets pairs is equal to the number of missing is-a relations in each iteration. For BioTop there are additionally the Source-Target pairs related to solutions based on ∃-expressions. The numbers for BioTop were 50, 62, 63 and 53 for the four iterations, respectively. The sizes for the Source and Target sets for the different iterations were small for most cases with sizes up to 10. This means that it is easy to visualize these sets in the system and the cognitive effort for the user is not so high. For some sets there were too many elements to have a suitable visualization in the current system.

Currently, the system removes redundant is-a relations from a solution after every iteration. This step is crucial for producing skyline optimal solutions. The advantage of removing redundant relations is the reduction of computation time as well as the reduction of unneccesary user interaction. However, in some cases redundancy may be interesting. For instance, developers may want to have explicitly stated is-a relations in the ontologies even though they are redundant. This can happen, for instance, for efficiency reasons in applications or as domain experts have validated asserted relations, these may be considered more trusted than derived relations. In this case, the minimality criterion is not considered important and we may aim for semantically maximal solutions. Our algorithms can be adapted by removing the redundancy checking. The algorithms would then try to find solutions at an as high level of informativeness, but not take into account redundancy. Also for finding solutions it may be interesting to keep redundancy. For instance, in situations where an is-a relation is repaired by a relation acquired from the axioms containing ∃ expressions it might be advantageous to keep also the missing is-a relation in subsequent iterations even though it is redundant. The reason for this is that the Source set and the Target set for the missing is-a relation might get updated in later iterations and therefore new repairing actions might be identified. One way to solve this is to make it possible in the system to show these missing is-a relations with their Source and Target sets but not to include them in the solution unless they are repaired using new knowledge. For example, let us assume that the missing is-a relation Human $\sqsubseteq $ Primate was repaired in one iteration by a repairing action {Human $\sqsubseteq $ Primate, SpeciesHomoSapiensQuality $\sqsubseteq $ OrderPrimatesQuality} in which case the second relation was found using ∃. In the next iteration the relation GreatApe $\sqsubseteq $ Primate was added to the ontology. If the system removed redundant relation Human $\sqsubseteq $ Primate then relation Human $\sqsubseteq $ GreatApe would not be detected as a possible repairing action for Human $\sqsubseteq $ Primate.

We note that our algorithms in every iteration except the last produce a skyline optimal solution that is on a higher level of informativeness than the solution in the previous iteration. This means that we get closer to a maxmin solution in every step. However, maxmin solutions are not guaranteed. Also, checking whether the solution in the final iteration is a maxmin solution would require full knowledge which we in general do not have and can only be obtained by a, for large ontologies, unfeasible brute-force method. This problem is inherent in GTAP^i^.

There are several factors that influence the performance of our algorithms. Some of these can, in principle, not be controlled. A first issue has to do with the domain expert. We assume that the domain expert answers correctly, but this is not sure. We assume that the missing is-a relations have been validated, but also here mistakes could have been made. Further, we assume that the original ontology is correct. For flat ontologies (few levels in the is-a hierarchy) our algorithms will repair the missing structure, but the possibility of finding more informative solutions is higher when the area around the missing is-a relations is not flat. How flat the original ontology is depends on the domain as well as the original ontology development. Our approaches find solutions that contain ŚcontributingŠ is-a relations, i.e., they will not compute solutions for which some is-a relations in the solution do not help explain the repairing of the missing is-a relations.

Our approach assumes that the ontologies are represented in description logics. The advantage of this approach is that we can use the formal tools of logic to generate solutions as well as that we are able to prove properties about the problem (e.g. complexity, existence of solutions) and the algorithms (e.g. soundness, properties of the generated solutions). Although more and more ontologies can be represented as logic-based ontologies, this may not be the case for all. Our system can still be used for such ontologies that contain a hierarchical structure, but there is no guarantee for the quality of the output.

Further, we note that the ’is-a relation’ is still not well-understood and/or used. For instance, [35] analyzed links in semantic networks and identified set/superset, generalization/specialization (based on predicates), ’a kind of’, and conceptual containment (related to lambda-abstraction) as different uses of ‘is-a’ and in [36] genus-subsumption, determinable-subsumption, specification and specialization were proposed. The problem of ‘is-a’ overloading is also addressed in [17]. Different uses of ‘is-a’ may not have the same properties. For instance, multiple inheritance does not make sense for all uses of ‘is-a’. These difficulties are not always recognized by ontology builders, while some may decide to focus one use of ‘is-a’. For instance, the Relation Ontology [37] for OBO defined the is-a relation for OBO ontologies, but is now superseeded by RO [38] in which no more definition for is-a is given, but instead the subclass construct of OWL is used. The work in this paper is based on logic and we assume that the is-a relation is reflexive, antisymmetric and transitive. The repairing of missing is-a relations in our work is based on logical reasoning. Our debugging tool does not take into account different uses of ’is-a’. Instead, it provides support for repairing missing structure that logically follows from decisions that were made by the developers of the ontologies.

For our algorithms we assume that the ontology extended with the missing is-a relations (*T*∪*M*) is consistent. This is important for ${\mathcal {EL}^{++}}$ ontologies as otherwise there is no solution. If *T*∪*M* is not consistent, we should first use approaches for debugging semantic defects^j^. Further, we assume for the algorithms that the missing is-a relations are validated. If these are not validated there is a risk that we introduce modeling defects in our ontologies.

For our OAEI Anatomy experiment we used sets of missing is-a relations that were generated by using an alignment between the two ontologies. Using an alignment allows us to generate missing is-a relations that are logically derivable from the information in the ontologies and the alignment. Our system can, in addition, also find missing is-a relations that were not logically derivable. This is the case whenever a missing is-a relation is repaired by using ‘new relations’ (Tables 4, 5, 6, 7 and 8). Further, we note that even though the alignment that was used is a reference alignment that has been used for many years, this alignment may still not be complete nor correct^k^. Therefore, even using the best ontology alignment systems may not provide us with complete alignments. Further, high-quality alignments may not always be available.

When alignments are available there could, however, be interesting ways of interaction between ontology alignment and ontology debugging. In [39] ontology alignment is considered as a special case of ontology debugging that focuses on completing the set of mappings between ontologies. A framework was proposed that unifies the phases of alignment and debugging and integrates them within one workflow. It is shown that debugging of the ontologies allows for improvement of the result of the alignment algorithms and vice versa.

The quality of the oracle also influences the quality of the repaired ontologies. In [30] different types of domain expert were discussed. The ’complete knowledge’ expert always answers the question whether an is-a relation is correct or not according to the domain in a correct manner. This is the desired case, but may not be always achievable. (People make mistakes and domain experts may not always agree.) The ’partial correct’ expert always gives correct answers, but may sometimes not give an answer. This represents a domain expert who knows a part of the domain well, but not the whole domain. To approximate this case we could use several domain experts and a skeptical approach. The ’Wrong’ expert may give wrong answers which implies that defects may be introduced in the ontologies. The use of tools such as the one presented in this paper will, however, reduce the introduction of errors in the ontology by the domain expert.

## Related work

There is not much work on the *completing of missing is-a structure*. In [19,34] this was addressed in the setting of taxonomies where the problem as well as some preference criteria were defined. Further, an algorithm was given and an implemented system was proposed. We note that the algorithm presented in this paper can be restricted to taxonomies and in that case finds more informative solutions than [19]. A later version of the [19] system, presented in [21], also deals with semantic defects, and was used for debugging ontologies related to a project for the Swedish National Food Agency [20]. An extension dealing with both ontology debugging and ontology alignment is described in [39]. In [40] an algorithm was given for finding solutions for ${\mathcal ALC}$ acyclic terminologies. In terms of the framework presented in this paper, those systems all returned solutions for GTAP, but there was no guarantee that the solutions were skyline optimal. Further, other heuristics were used.

There is no other work yet on *GTAP*. There is some work on TBox abduction. Hubauer et al. [41] proposes an automata-based approach to TBox abduction in $\mathcal {EL}$. It is based on a reduction to the axiom pinpointing problem which is then solved with automata-based methods.

Further, there is work that addresses *related topics* but not directly the problem that is addressed in this paper.

*Detection of missing (is-a) relations:* In [14] the authors propose an approach for detecting modeling and semantic defects within an ontology based on patterns and antipatterns. The patterns and antipatterns are logic-based and mainly deal with logical constructs not available in taxonomies. Some suggestions for repairing are also given. In [18-21] detection is preformed using the mappings between two ontologies. Given two pairs of terms between two ontologies which are linked by the same kind of relationship, if the two terms in one ontology are linked by an is-a relation while the corresponding terms in the other are not, then a candidate missing is-a relation is detected. The work in [16] discusses the alignment of AMA and NCI-A and uses the notion of structural validation to remove mappings that cannot be structurally validated. Structural validation could be used to detect candidate missing is-a relations.

The properties of is-a can be used for detecting modeling defects. For instance, based on the notions of identity, rigidity and dependence, not all is-a relations in existing ontologies make sense [17]. These is-a relations can be detected by checking these properties. In [15] two reasoning services are proposed for detecting flaws in OWL property expressions. The defects relate to the property is-a hierarchy, domain and range axioms and property chains.

Detecting missing is-a relations may be seen as a special case of detecting relations. There is much work on finding relationships between terms in the ontology learning area [11]. In this setting, new ontology elements are derived from text using knowledge acquisition techniques. There is, however, also work specifically focused on the discovery of is-a relations. One paradigm is based on linguistics using lexico-syntactic patterns. The pioneering research conducted in this line is in [13], which defines a set of patterns indicating is-a relationships between words in the text. However, depending on the chosen corpora, these patterns may occur rarely. Thus, though the approach has a reasonable precision, its recall is very low. Other linguistic approaches may make use of, for instance compounding, the use of background and itemization, term co-occurrence analysis or superstring prediction (e.g. [42,43]). Another paradigm is based on machine learning and statistical methods, such as k-nearest neighbors approach [23], association rules [22], bottom-up hierarchical clustering techniques [25], supervised classification [26] and formal concept analysis [24]. Ontology evolution approaches [12,44] allow for the study of changes in ontologies and using the change management mechanisms to detect candidate missing relations.

As mentioned before, these approaches, in general, do not detect all missing is-a relations.

*Debugging of semantic defects:* There is much work on debugging of semantic defects which is a dual problem to the one addressed in this paper. Most of the work on this topic aims at identifying and removing logical contradictions from an ontology [21,45-49], from mappings between ontologies [21,50-53] or ontologies in a network [20,21,54]. There is more work that addresses semantic defects in ontologies. Most of it aims at identifying and removing logical contradictions from an ontology. Standard reasoners are used to identify the existence of a contradiction, and provide support for resolving and eliminating it [49]. In [46] minimal sets of axioms are identified which need to be removed to render an ontology coherent. An algorithm for finding solutions is proposed which uses a variant of the single relation heuristic. Similarly, in [47,48] strategies are described for repairing unsatisfiable concepts detected by reasoners, explanation of errors, ranking erroneous axioms, and generating repair plans. The generated solutions, however, are based on other heuristics than [21,46]. In [45] the focus is on maintaining the consistency as the ontology evolves through a formalization of the semantics of change for ontologies. In [50-52] the setting is extended to repairing ontologies connected by mappings. In this case, semantic defects may be introduced by integrating ontologies. All approaches assume that ontologies are more reliable than the mappings and try to remove some of the mappings to restore consistency. In [50,52] the solutions are based on the computation of minimal unsatisfiability-preserving sets or minimal conflict sets. While [50] proposes solutions based on a heuristic using distance in WordNet, [52] allows the user to choose between all, some or one solution. In [51] the authors focus on the detection of certain kinds of defects and redundancy. The work in [53] further characterizes the problem as mapping revision. Using belief revision theory, the authors give an analysis for the logical properties of the revision algorithms. The approach in [54] deals with the inconsistencies introduced by the integration of ontologies, and unintended entailments validated by the user. We note that most of these approaches can deal with ontologies represented in more expressive languages than in our work. However, few of the early approaches have implemented systems and were usually only tested on small ontologies. Recently, several ontology alignment systems such as LogMap and AML manage to produce alignments with a low incoherence ratio for the Anatomy and the Large Biomedical Ontologies tracks of the OAEI (e.g. [55]). One remaining problem with these approaches is that the choice of which information to remove is completely logic-based and therefore may prefer solutions with modeling defects over solutions that are correct according to the domain [56].

*Abductive reasoning in (simple) description logics:* In addition to TBox abduction, [29] defines three more abduction problems. Concept abduction deals with finding sub-concepts. Abox abduction deals with retrieving instances of concepts or roles that, when added to the knowledge base, allow the entailment of a desired ABox assertion. Knowledge base abduction includes both ABox and TBox abduction. Most of the existing work deals with concept abduction and ABox abduction. The work on concept abduction is based on tableau-based (e.g. [57,58]) or structural subsumption (e.g. [59]) approaches. The work on Abox abduction often uses a tableau-based method (e.g. [60,61]) or an abductive logic programming approach (e.g. [62,63]). There is also work on the complexity of the ABox abduction (e.g. [64]) and concept abduction problems (e.g. [65]).

## Conclusions and future work

In this paper we presented an approach for completing the is-a structure of ${\mathcal {EL}}$ and ${\mathcal {EL}^{++}}$ ontologies. Many biomedical ontologies can be represented by ${\mathcal {EL}}$ or a small extension thereof. We first defined a model of GTAP and extended it with various preferences. Then we presented complexity results on the existence, relevance and necessity decision problems for ontologies that can be represented as TBoxes using a member of the ${\mathcal {EL}}$ family. Unless the polynomial hierarchy collapses, GTAP is much harder than the classical deduction problem, which is tractable for ${\mathcal {EL}^{++}}$. Further, we provided algorithms and a system for finding skyline optimal solutions to the GTAP, and evaluated our approach on three biomedical ontologies. The evaluation has shown the usefulness of the system as in all experiments new is-a relations have been identified.

In the future, we are interested in studying the GTAP for other knowledge representation languages. Further, we will investigate variants of the GTAP with different preference relations and restrictions of the signature. Another interesting topic is to study the GTAP in the context of modular ontologies where it may not be possible to introduce changes in the imported ontologies. Further, we will look into the integration of different abduction frameworks to deal with both modeling and semantic defects.

## Endnotes

^a^ As an example, for SNOMED all constructors are in ${\mathcal {EL}^{++}}$. Also taxonomies can be represented in ${\mathcal {EL}}$. Gene Ontology has, in addition to ${\mathcal {EL}}$ constructs, some inverse roles and NCI Thesaurus has some disjunctions. We note that, although our approaches do not consider constructors outside ${\mathcal {EL}^{++}}$, our algorithms still will find correct solutions for these ontologies. Further, to deal with more expressive languages other less efficient techniques may be necessary such as in [40] where a tableau-based method is used for ${\mathcal ALC}$ acyclic terminologies. Another case is MeSH which is a thesaurus, but the hierarchical relation does not always express is-a, and therefore, although the algorithms can be applied to MeSH, the proposed solutions may not always be logically correct.

^b^ PubMed accessed on 21-02-2014.

^c^ Therefore, the approach in this paper can also be seen as a detection method that takes already found missing is-a relations as input.

^d^ Observe that both missing is-a relations are derivable using S _1_. GranulomaProcess $\sqsubseteq $ NonNormalProcess is derivable as GranulomaProcess $\sqsubseteq $ InflammationProcess (S _1_), InflammationProcess $\sqsubseteq $ PathologicalProcess (S _1_), and PathologicalProcess $\sqsubseteq $ NonNormalProcess (*T*). Endocarditis $\sqsubseteq $ PathologicalPhenomenon is derivable as Endocarditis $\sqsubseteq \exists $hasAssociatedProcess.InflammationProcess (*T*), ∃hasAssociatedProcess.InflammationProcess $\sqsubseteq \exists $hasAssociatedProcess.PathologicalProcess (S _1_), and ∃hasAssociatedProcess.PathologicalProcess $\sqsubseteq $ PathologicalPhenomenon (*T*).

^e^ For an ontology of 3000 concepts (similar in size as the ontologies in our OAEI Anatomy experiments) this method would need to ask the domain expert 9000000 questions. With a smart strategy this number can be reduced a lot. For instance, if we know that limb joint is a joint, then we also know that every subconcept of limb joint is a joint and thus we do not need to ask the domain expert. However, even if we can reduce the search space by 90% we would still need to ask the domain expert 900000 questions. This is not feasible. We also note that this brute-force method is essentially ontology development.

^f^ The algorithm without lines 14-15 provides a *RepairSingleIsa* for taxonomies.

^g^ Our aim is that a domain expert with ontology engineering expertise can use tools based on our approach without much introduction. If the domain expert lacks this expertise, an ontology engineer may work together with the domain expert. The domain expert needs to make the decisions on the validity of is-a relations, while the ontology engineer may help with understanding is-a (e.g. as opposed to part-of) and understanding the consequences of a particular repairing. In an earlier experiment for the Swedish National Food Agency [20] the domain expert had some expertise in ontology engineering and few help from us was needed.

^h^ An optimized version of this approach is shown in [34].

^i^ This relates also to the difference between the classic abduction problem where solutions can be constructed starting from *H*, while we can only validate solutions in GTAP using *Or*.

^j^ A system that integrates completing of ontologies with debugging of semantic defects for taxonomies is presented in [21].

^k^ In [21] it is suggested that 12 mappings in the alignment are not correct.

## Appendix - complexity proofs

In this appendix we prove the complexity results shown in Tables 2 and 3.

The proof for the existence problem for the general case of GTAP follows the technique presented in Theorem 5.2 of [27]. In general, the existence problem is not harder than the relevance problem.

Since it holds that every definite Horn theory can be represented by a general ${\mathcal {EL}}$ TBox and every Horn theory can be represented by a general ${\mathcal {EL}^{++}}$ TBox [65], some existing complexity results on the abduction of Horn theory can be adapted here for the case of general existence and subset minimality case. Note that this applies to the hardness proofs.

For convenience we primarily deal with dispensability rather than with necessity. Results for necessity are easy corollaries to our results on dispensability. **Dispensability** Given *ψ*, does a solution $S \in {\mathcal {S}}(T, C, Or$, *M*) exist such that *ψ*∉*S*?

### ᅟ

#### Complexity - ${\mathcal {EL}^{++}}$

##### General case

###### Theorem 1.

To decide if ${\mathcal {S}}(T, C, Or, M) \neq \emptyset $ for a given GTAP (*T*,*C*,*Or*,*M*) is NP-complete.

###### *Proof*.

The entailment problem of ${\mathcal {EL}^{++}}$ is tractable [6]. Therefore the membership in NP follows.

NP-hardness of this problem is shown by a transformation from well-known satisfiability problem (SAT), cf. [66]. Let *Cl*={*Cl*_1_,…,*Cl*_*m*_} be a set of propositional clauses on *X*={*x*_1_,…,*x*_*n*_}. Let *X*^′^={*x*1′,…,*xn*′}, *G*={*g*_1_,…,*g*_*m*_}, *R*={*r*_1_,…,*r*_*n*_} be sets of new concepts and *c* be a new concept. Then, the GTAP (*T*,*C*,*Or*,*M*) is constructed as follows.

Note that in order to simplify the presentation, for the definition of the oracle, we write *Or* as a set containing the subsumptions that are *true* according to the oracle. We also apply this simplification in the other proofs of the paper.

$$\begin{aligned} C &= X \cup X' \cup G \cup R \cup c \\ M &= \{ c {\sqsubseteq} r_{i}: 1 \leq i \leq n, ~~ c {\sqsubseteq} g_{j} : 1 \leq j \leq m \} \\ Or &= \{ c {\sqsubseteq} x_{i}: 1 \leq i \leq n, ~~ c {\sqsubseteq} x^{\prime}_{i} : 1 \leq j \leq n \} \\ T &= \{ x_{i} \sqcap x^{\prime}_{i} {\sqsubseteq} \bot, x_{i} {\sqsubseteq} r_{i}, x'_{i} {\sqsubseteq} r_{i} : 1 \leq i \leq n \} \cup \{ c {\sqsubseteq} \top, \top {\sqsubseteq} c\}\\ & \bigcup\limits_{i=1}^{m} {\left(\{ x_{j} {\sqsubseteq} g_{i} : x_{j} \in {Cl}_{i} \} \cup \{ x^{\prime}_{j} {\sqsubseteq} g_{i} : \neg x_{j} \in {Cl}_{i} \}\right)} \end{aligned} $$

Next we prove that *Cl* is satisfiable iff (*T*,*C*,*Or*,*M*) has a solution. We first observe that for each $S \in {\mathcal {S}}(T,C,Or,M)$, either $c {\sqsubseteq } x_{i} \in S$ or $c {\sqsubseteq } x'_{i} \in S$ (but not both) must hold, for 1≤*i*≤*n*, since otherwise $T \cup S \not \models c {\sqsubseteq } r_{i}$.

Assume *Cl* is satisfiable. Let *ψ* be the truth assignment such that *ψ*(*Cl*) is *true*. Define the solution *S* as

$$\begin{aligned} S & = \{ c {\sqsubseteq} x_{i} : \psi(x_{i}) = true, 1 \leq i \leq n \} \cup \\ &\quad\,\, \{ c {\sqsubseteq} x^{\prime}_{i} : \psi(x_{i}) = false, 1 \leq i \leq n \} \end{aligned} $$

Then $T \cup S \models c {\sqsubseteq } r_{1} \wedge \ldots \wedge c {\sqsubseteq } r_{n}$. Moreover, because for every *Cl*_*i*_(1≤*i*≤*m*)*ψ*(*Cl*_*i*_) is *true*, we have $T \cup S \models c {\sqsubseteq } g_{1} \wedge \ldots \wedge c {\sqsubseteq } g_{m}$. Therefore *T*∪*S*⊧*M* holds.

Consider *Cl* is not satisfiable. For a solution *S*, either *x*_*i*_ or $x^{\prime }_{i}$ must exist in *S*. Since there does not exist any truth assignment such that *ψ*(*Cl*) is *true*, there does not exist such *S* such that $T \cup S \models c {\sqsubseteq } g_{1} \wedge \ldots \wedge c {\sqsubseteq } g_{m}$. Therefore ${\mathcal {S}}(T, C, Or, M) = \emptyset $.

♣ □

###### Theorem 2.

To decide if a given *ψ* is relevant for a given GTAP (*T*,*C*,*Or*,*M*) is NP-complete. To decide if a given *ψ* is dispensable for a given GTAP (*T*,*C*,*Or*,*M*) is NP-complete.

###### *Proof*.

Guess a solution *S* which contains *ψ* (resp. does not contain *ψ*). Since the checking if $S \in {\mathcal {S}}(T, C, Or, M)$ is in P, the membership in NP follows.

Hardness can be proven by a slight modification of the reduction for the existence problem in Theorem 1. Define the GTAP (*T*^′^,*C*^′^,*Or*^′^,*M*^′^) as

$$\begin{aligned} C^{\prime}& = C \cup e \cup e^{\prime} \\ M^{\prime} & = M \cup h \\ Or^{\prime} & = Or \cup \{c {\sqsubseteq} e, c {\sqsubseteq} e^{\prime}\} \\ T^{\prime} & = T \setminus \{x_{i} {\sqsubseteq} r_{i}, x^{\prime}_{i} {\sqsubseteq} r_{i} : 1 \leq i \leq n \} \cup \\ &\quad \{x_{i} \sqcap e {\sqsubseteq} r_{i}, x^{\prime}_{i} \sqcap e {\sqsubseteq} r_{i} : 1 \leq i \leq n \} \cup \\ &\quad \{ e^{\prime} {\sqsubseteq} r_{i}: 1 \leq i \leq n, ~~ e^{\prime} {\sqsubseteq} g_{j} : 1 \leq j \leq m \} \cup \\ &\quad\{e \sqcap e^{\prime} {\sqsubseteq} \bot, e {\sqsubseteq} h, e^{\prime} {\sqsubseteq} h\} \end{aligned} $$

where *e*,*e*^′^,*h* are new concepts not occurring in *C*.

We show that *Cl* is satisfiable if and only if (*T*^′^,*C*^′^,*Or*^′^,*M*^′^) has a solution containing $c {\sqsubseteq } e$ and not containing $c {\sqsubseteq } e'$.

Assume *Cl* is satisfiable. Let *ψ* be the truth assignment such that *ψ*(*Cl*) is *true*. Define the solution *S* as

$$\begin{array}{lll} S & = &\{ c {\sqsubseteq} x_{i} : \psi(x_{i}) = true, 1 \leq i \leq n \} \cup \\ &&\{ c {\sqsubseteq} x^{\prime}_{i} : \psi(x_{i}) = false, 1 \leq i \leq n \} \cup \{c {\sqsubseteq} e\} \end{array} $$

Then *T*^′^∪*S*⊧*M*^′^ holds. Note that one and only one of $c {\sqsubseteq } e$ and $c {\sqsubseteq } e^{\prime }$ is in any solution to (*T*^′^,*C*^′^,*Or*^′^,*M*^′^). Therefore, $c {\sqsubseteq } e^{\prime } \not \in S$ holds.

Assume *Cl* is not satisfiable. Then the solution *S* is $\{ c {\sqsubseteq } e^{\prime }\}$. Then $c {\sqsubseteq } e \not \in S$ holds. This concludes the proof.

♣ □

##### Subset minimality

###### Theorem 3.

To decide if ${\mathcal {S}}_{\textit {min}}(T, C, Or, M) \neq \emptyset $ for a given GTAP (*T*,*C*,*Or*,*M*) is NP-complete.

###### *Proof*.

We show that the problem is equivalent to the existence problem in general case. That is, ${\mathcal {S}}_{\textit {min}}(T, C, Or, M) \neq \emptyset $ iff ${\mathcal {S}}(T, C, Or, M) \neq \emptyset $. The ’only if’ direction is trivial. Now we prove the ’if’ direction. We show that if there is a solution $S \in {\mathcal {S}}(T, C, Or, M)$, then there is a solution $S^{\prime } \in {\mathcal {S}}_{\textit {min}}(T, C, Or, M)$ and *S*^′^⊆*S*. If *S* is subset minimal, then *S*^′^=*S*. Otherwise, let  be the set of all solutions *S*^′′^ such that *S*^′′^⊂*S*. Since the empty set is not a solution, there exists an $S^{\prime } \in \mathcal {W}$, such that $\forall P \in \mathcal {W}$, *P*⊄*S*^′^ holds. Then *S*^′^ is a subset minimal solution.

♣ □

###### Theorem 4.

To decide if a given *ψ* is min-relevant for a given GTAP (*T*,*C*,*Or*,*M*) is NP-complete. To decide if a given *ψ* is min-dispensable for a given GTAP (*T*,*C*,*Or*,*M*) is NP-complete.

###### *Proof*.

Membership: guess a set *S* which contains (resp. does not contain) *ψ*. Note that $S \in {\mathcal {S}}_{\textit {min}}(T, C, Or, M)$ iff $S \in {\mathcal {S}}(T, C, Or, M)$ and $\forall h \in S: S \setminus \{h\} \not \in {\mathcal {S}}(T, C, Or, M)$. This is due to the monotonicity of ⊧ in ${\mathcal {EL}^{++}}$. The checking is in P, hence the membership in NP follows.

Hardness under the restrictions follows immediately by Theorem 2.

♣ □

##### Semantic maximality

###### Theorem 5.

To decide if ${\mathcal {S}}^{max}(T, C, Or, M) \neq \emptyset $ for a given GTAP (*T*,*C*,*Or*,*M*) is NP-complete.

###### *Proof*.

The proof is analogous to that of Theorem 3: we show that the problem is equivalent to the existence problem of the general case. That is, ${\mathcal {S}}^{max}(T, C, Or, M) \neq \emptyset $ iff ${\mathcal {S}}(T, C, Or, M) \neq \emptyset $. The ’only if’ direction is trivial. Now we prove the ’if’ direction. We show that if there is a solution $S \in {\mathcal {S}}(T, C, Or, M)$, then there is a solution $S^{\prime } \in {\mathcal {S}}^{max}(T, C, Or, M)$ and *S*⊆*S*^′^. Let  be the set of all solutions *S*^′′^ that *S*⊆*S*^′′^. Then there exists $S^{\prime } \in \mathcal {W}$, such that $\forall P \in \mathcal {W}$, *S*^′^⊄*P* holds. It is easy to show that *S*^′^ is semantically maximal. Assume the opposite. There is another solution *S*_1_ which is more informative than *S*^′^. That is, there is a *ψ* such that *T*∪*S*_1_⊧*S*^′^∪{*ψ*} and *T*∪*S*^′^⊮*ψ*. Then *S*^′^∪*S*_1_ should be a solution and it is a superset of *S*^′^. ⇒ Contradiction.

♣ □

###### Theorem 6.

To decide if a given *ψ* is max-relevant for a given GTAP (*T*,*C*,*Or*,*M*) is NP-complete. To decide if a given *ψ* is max-dispensable for a given GTAP (*T*,*C*,*Or*,*M*) is NP-complete.

###### *Proof*.

Membership: guess a set *S* which contains (resp. does not contain) *ψ*. $S \in {\mathcal {S}}^{max}(T, C, Or, M)$ iff $S \in {\mathcal {S}}(T, C, Or, M)$ and ∀*h*∈*Or* s.t. *T*∪*S*⊮*h*:*T*∪*S*∪{*h*}⊧*M*. This is due to the monotonicity of ⊧ in ${\mathcal {EL}^{++}}$. The checking can be done in polynomial time since the number of possible TBox assertions is polynomial to *C*. Hence the membership follows.

Hardness under the restrictions follows immediately by Theorem 2.

♣ □

##### Skyline

Due to the fact that the set of skyline optimal solutions contains all subset minimal solutions, the existential problem follows trivially. That is, if there exists a subset minimal solution, then there exists a skyline optimal solution.

###### Theorem 7.

To decide if ${\mathcal {S}}_{\textit {min}}^{max}(T, C, Or, M) \neq \emptyset $ for a given GTAP (*T*,*C*,*Or*,*M*) is NP-complete.

###### Theorem 8.

To decide if a given *ψ* is skyline-relevant for a given GTAP (*T*,*C*,*Or*,*M*) is NP-complete. To decide if a given *ψ* is skyline-dispensable for a given GTAP (*T*,*C*,*Or*,*M*) is NP-complete.

###### *Proof*.

Membership: guess a set *S* which contains (resp. does not contain) *ψ*. Note that $S \in {\mathcal {S}}_{\textit {min}}^{max}(T, C, Or, M)$ iff $S \in {\mathcal {S}}(T, C, Or, M)$ and ∀*h*∈*S*:*T*∪(*S*∖{*h*})⊮*S*. This is due to the monotonicity of ⊧ in ${\mathcal {EL}^{++}}$. The checking is in P, hence the membership in NP follows. Hardness under the restrictions follows immediately by Theorem 2.

♣ □

##### Maxmin

###### Theorem 9.

To decide if ${\mathcal {S}}_{\textit {min}}^{\textbf {max}}(T, C, Or, M) \neq \emptyset $ for a given GTAP (*T*,*C*,*Or*,*M*) is NP-complete.

###### *Proof*.

Again, we show that the problem is equivalent to the existence problem of the general case. Since the existence problem of ${\mathcal {S}}^{max}(T, C, Or, M)$ is shown to be equivalent to the general case, there exists ${\mathcal {S}}^{max}(T, C, Or, M)$. Since ${\mathcal {S}}_{\textit {min}}^{\textbf {max}}(T, C, Or, M) \subseteq {\mathcal {S}}^{max}(T, C, Or, M)$ holds, we need to remove from ${\mathcal {S}}^{max}(T, C, Or, M)$ those solutions {*S*|∃*S*^′^,*s*.*t*.*S*^′^⊂*S*:*T*∪*S*^′^⊧*S*}. Given a maximal solution *S*, we call such an *S*^′^ the witness of *S*. Note that if $S \in {\mathcal {S}}^{max}(T, C, Or, M)$, then all the witnesses of *S* as defined above are also in ${\mathcal {S}}^{max}(T, C, Or, M)$. Therefore, during the removing process, if *S* is removed, *S* must have a witness *S*^′^ and *S*^′^ is still in ${\mathcal {S}}^{max}(T, C, Or, M)$. As a result, there will be at least one solution remaining in ${\mathcal {S}}^{max}(T, C, Or, M)$ after the removal process. This concludes the proof.

♣ □

###### Theorem 10.

To decide if a given *ψ* is maxmin-relevant for a given GTAP (*T*,*C*,*Or*,*M*) is NP-complete. To decide if a given *ψ* is maxmin-dispensable for a given GTAP (*T*,*C*,*Or*,*M*) is NP-complete.

###### *Proof*.

Membership: guess a set *S* which contains (resp. does not contain) *ψ*. Note that $S \in {\mathcal {S}}_{\textit {min}}^{\textbf {max}}(T, C, Or, M)$ iff $S \in {\mathcal {S}}^{max}(T, C, Or, M)$ and ∀*h*∈*S*:*T*∪(*S*∖{*h*})⊮*S*.

To check whether $S \in {\mathcal {S}}^{max}(T, C, Or, M)$ is feasible in polynomial time as shown in Theorem 6. The minimality check is also feasible in polynomial time as shown in Theorem 8, hence the membership in NP follows. Hardness under the restrictions follows immediately by Theorem 2.

♣ □

##### Minmax

###### Theorem 11.

To decide if ${\mathcal {S}}_{\textbf {min}}^{max}(T, C, Or, M) \neq \emptyset $ for a given GTAP (*T*,*C*,*Or*,*M*) is NP-complete.

###### *Proof*.

We show that the problem is equivalent to the existence problem of the general case. That is, ${\mathcal {S}}_{\textbf {min}}^{max}(T, C, Or, M) \neq \emptyset $ iff ${\mathcal {S}}(T, C, Or, M) \neq \emptyset $. If there is a solution $S \in {\mathcal {S}}(T, C, Or, M)$, then from Theorem 3 there is a solution which is subset minimal. Let  be the set of all the subset minimal solutions. Then we remove from  the solutions which are less informative, in the sense that if there is $S^{\prime }, S^{\prime \prime } \in \mathcal W$ such that *S*^′^ is more informative than *S*^′′^, then *S*^′′^ is removed. Since the relation *more informative* is transitive, the removal process is confluent. Then there exists a unique non-empty set $\mathcal W^{\prime } \subseteq \mathcal W$, such that no solution is more informative than another. It is obvious that $\mathcal W^{\prime }$ is ${\mathcal {S}}_{\textbf {min}}^{max}(T, C, Or, M)$.

♣ □

###### Theorem 12.

To decide if a given *ψ* is minmax-relevant for a given GTAP (*T*,*C*,*Or*,*M*) is ${\Sigma _{2}^{P}}$-complete. To decide if a given *ψ* is minmax-dispensable for a given GTAP (*T*,*C*,*Or*,*M*) is ${\Sigma _{2}^{P}}$-complete.

###### *Proof*.

Membership can be shown by first guessing a solution *S* containing (resp. not containing) *ψ*, then verifying if $S \in {\mathcal {S}}_{\textbf {min}}^{max}(T, C, Or, M)$. That is, to check whether there does not exist a subset minimal solution which is more informative than *S*. The check can be done by a co-NP oracle, since checking that there does exist such a solution can be done in NP (we guess a solution *S*^′^. Checking *S*^′^ is subset minimal and *S*^′^ is more informative than *S* can be done in polynomial time). Therefore, the membership in ${\Sigma _{2}^{P}}$ follows.

${\Sigma _{2}^{P}}$-hardness of this problem is shown by a transformation from deciding *Φ*∈ QBF _2,∃_. Let *Φ* without loss of generality be a QBF ∃*x*_1_…∃*x*_*n*_∀*y*_1_…∀*y*_*m*_*E*. Let *E* be in disjunctive normal form *D*_1_∨⋯∨*D*_*l*_ where *D*_*i*_(1≤*i*≤*l*) is a conjunction of literals. Let *X*={*x*_1_,…,*x*_*n*_}, *Y*={*y*_1_,…,*y*_*m*_}, $X^{\prime } = \{x^{\prime }_{1}, \ldots, x^{\prime }_{n}\}$, and $Y^{\prime } = \{y^{\prime }_{1}, \ldots, y^{\prime }_{m}\}$. Let further *G*={*g*_1_,…,*g*_*m*_}, *R*={*r*_1_,…,*r*_*n*_} be sets of new concepts and *h*, *e*, *e*^′^, *c* be new concepts. Then, the GTAP (*T*,*C*,*Or*,*M*) is constructed as follows.

$$\begin{aligned} C & = X \cup X^{\prime} \cup Y \cup Y^{\prime} \cup G \cup R \cup h \cup c \cup e \cup e^{\prime}\\ M & = \{ c {\sqsubseteq} h\}\\ Or & = \{ c {\sqsubseteq} e, c {\sqsubseteq} e^{\prime}, c {\sqsubseteq} x_{i}: 1 \leq i \leq n, \\ & \quad\, c {\sqsubseteq} x^{\prime}_{i}: 1 \leq i \leq n, c {\sqsubseteq} y_{j}: 1 \leq j \leq m, \\ & \quad\, c {\sqsubseteq} y^{\prime}_{j}: 1 \leq j \leq m\}\\ \end{aligned} $$

$$\begin{aligned} T & = \{c{\sqsubseteq} \top, \top {\sqsubseteq} c\} \\ & \cup \{ x_{i} \sqcap x^{\prime}_{i} {\sqsubseteq} \bot, x_{i} \sqcap e {\sqsubseteq} r_{i}, x^{\prime}_{i} \sqcap e {\sqsubseteq} r_{i} : 1 \leq i \leq n \} \\ & \cup \{ r_{1} \sqcap \ldots \sqcap r_{n} {\sqsubseteq} h\} \\ & \cup \{ y_{i} \sqcap y^{\prime}_{i} {\sqsubseteq} \bot, y_{i} \sqcap e^{\prime} {\sqsubseteq} g_{i}, y^{\prime}_{i} \sqcap e^{\prime} {\sqsubseteq} g_{i} : 1 \leq i \leq m \} \\ & \cup \{g_{1} \sqcap \ldots \sqcap g_{m} {\sqsubseteq} e\} \cup T^{\prime} \cup T^{\prime\prime}\\ \end{aligned} $$

$$\begin{aligned} T^{\prime} & = \bigcup\limits_{i=1}^{l} \bigcup\limits_{j=1}^{s} {\big(\{ y_{i_{1}} \sqcap \ldots \sqcap y_{i_{p}} \sqcap y^{\prime}_{i_{p+1}} \sqcap \ldots \sqcap y^{\prime}_{i_{q}}} \\ & \quad \sqcap_{k=1, k\neq j}^{s} {x_{i_{k}}} \sqcap_{k=s+1}^{t} {x^{\prime}_{i_{k}}} ~~~ {\sqsubseteq} ~~~ x^{\prime}_{i_{j}}: \\ D_{i} &= y_{i_{1}} \wedge \ldots \wedge y_{i_{p}} \wedge \neg y_{i_{p+1}} \wedge \ldots \wedge \neg y_{i_{q}} \\ & \quad\wedge x_{i_{1}} \wedge \ldots \wedge x_{i_{s}} \wedge \neg x_{i_{s+1}} \wedge \ldots \wedge \neg x_{i_{t}} \}\big) \\ T^{\prime\prime}& = \bigcup\limits_{i=1}^{l} \bigcup\limits_{j=s+1}^{t} {\big(\{ y_{i_{1}} \sqcap \ldots \sqcap y_{i_{p}} \sqcap y^{\prime}_{i_{p+1}} \sqcap \ldots \sqcap y^{\prime}_{i_{q}}} \\ &\quad \sqcap_{k=1}^{s} {x_{i_{k}}} \sqcap_{k=s+1, k\neq j}^{t} {x^{\prime}_{i_{k}}} ~~~ {\sqsubseteq} ~~~ x_{i_{j}}: \\ D_{i} &= y_{i_{1}} \wedge \ldots \wedge y_{i_{p}} \wedge \neg y_{i_{p+1}} \wedge \ldots \wedge \neg y_{i_{q}} \\ &\quad \wedge x_{i_{1}} \wedge \ldots \wedge x_{i_{s}} \wedge \neg x_{i_{s+1}} \wedge \ldots \wedge \neg x_{i_{t}} \}\big) \end{aligned} $$

Intuitively, for each disjunct *D*_*i*_ in *E*, for each *x* literal in *D*_*i*_, *T*^′^ and *T*^′′^ consists of a subsumption where the negated form of *x* is at the right hand side. More precisely, if *x* is of the form *x*_*i*_, then $x^{\prime }_{i}$ occurs at the right hand side; if *x* is of the form ¬*x*_*i*_, then *x*_*i*_ occurs at the right hand side. For instance, assume *D*_*i*_=*y*_1_∧¬*y*_2_∧*x*_1_∧¬*x*_2_. Then *T*^′^ consists of the subsumption $y_{1} \sqcap y_{2}^{\prime } \sqcap x^{\prime }_{2} {\sqsubseteq } x^{\prime }_{1}$, and *T*^′′^ consists of $y_{1} \sqcap y_{2}^{\prime } \sqcap x_{1} {\sqsubseteq } x_{2}$.

Note that *T* is consistent and that (*T*,*C*,*Or*,*M*) is constructible in polynomial time. We show that *Φ*∈ QBF _2,∃_ holds iff ($c {\sqsubseteq } e) \in S$ (resp. ($c {\sqsubseteq } e^{\prime }) \not \in S$) such that $S \in {\mathcal {S}}_{\textbf {min}}^{max}(T, C, Or, M)$.

“Only if”: Assume *Φ*∈ QBF _2,∃_ holds. Hence, there exists a truth assignment *ϕ*(*X*) such that ∀*y*_1_…∀*y*_*m*_*E*_*ϕ*_(*X*)∈ QBF _1,∀_ holds. Define the solution *S* as

$S = \{ c {\sqsubseteq } x_{i} : \phi (x_{i}) = true, 1 \leq i \leq n \} \cup \{ c {\sqsubseteq } x^{\prime }_{i} : \phi (x_{i}) = false, 1 \leq i \leq n \} \cup \{ c {\sqsubseteq } e\}$.

Then *T*∪*S*⊧*M*. Moreover, *S* is subset minimal. Next we show there is no other subset minimal solution which is more informative than *S*. Other than *ϕ*, there are 2^*n*^−1 possible truth assignments over *X*. For each such truth assignment *ψ*, we can obtain the corresponding solution *S*^′^, analogously to the way obtaining *S* by replacing *ϕ* with *ψ*. Then every such *S*^′^ is a subset minimal solution. However, it is obvious that *T*∪*S*^′^⊮*S*, since *S*≠*S*^′^ and there is at least one variable *x*_*i*_ such that *ϕ*(*x*_*i*_)≠*ψ*(*x*_*i*_).

Let *μ* be an arbitrary truth assignment over *Y*. Define *S*^′^ as

$S^{\prime } = \{ c {\sqsubseteq } y_{i} : \mu (y_{i}) = true, 1 \leq i \leq m \} \cup \{ c {\sqsubseteq } y^{\prime }_{i} : \mu (y_{i}) = false, 1 \leq i \leq m \} \cup \{ c {\sqsubseteq } e^{\prime }\}$. Any other subset minimal solution *S*^′′^ which does not contain $c {\sqsubseteq } e$ must contain such an *S*^′^. Note that we do not fix *S*^′^ since *μ* is arbitrary. To prove *S* is a minmax solution, we need to show that there does not exist such a subset minimal solution *S*^′′^ such that *T*∪*S*^′′^⊧*S* holds. In the following we show that for every such a possible solution *S*^′′^, *T*∪*S*^′′^∪*S* is inconsistent.

Since ∀*y*_1_…∀*y*_*m*_*E*_*ϕ*_(*X*)∈ QBF _1,∀_ holds, there exists a disjunct *D*_*i*_∈*E*, such that *D*_*i*__*ϕ*,*μ*_(*X*,*Y*) is *true*. That is, for every *z*∈*D*_*i*_, $c {\sqsubseteq } z \in S \cup S^{\prime \prime }$ and for every ¬*z*∈*D*_*i*_, $c {\sqsubseteq } z^{\prime } \in S \cup S^{\prime \prime }$. Let *ρ* be a rule in *T*^′^∪*T*^′′^ regarding *D*_*i*_ (w. l. o. g.) with the form:

$$\begin{array}{l} y_{i_{1}} \sqcap \ldots \sqcap y_{i_{p}} \sqcap y^{\prime}_{i_{p+1}} \sqcap \ldots \sqcap y^{\prime}_{i_{q}} \\ \sqcap_{k=1, k\neq j}^{s} x_{i_{k}} \sqcap_{k=s+1}^{t} {x^{\prime}_{i_{k}}} {\sqsubseteq} x^{\prime}_{i_{j}} \end{array} $$

Since *ρ*∈*T*, we have $T \cup S^{\prime \prime } \cup S \models c {\sqsubseteq } x^{\prime }_{i_{j}}$. On the other hand, $T \cup S^{\prime \prime } \cup S \models c {\sqsubseteq } x_{i_{j}}$ holds too, because $x_{i_{j}} \in D_{i}$. Therefore *T*∪*S*^′′^∪*S* is not consistent, hence *T*∪*S*^′′^⊮*S*.

“If”: Assume *Φ*∈ QBF _2,∃_ does not hold. Hence, for every truth assignment *ϕ*(*X*), there exists a truth assignment *μ*(*Y*), such that *E*_*ϕ*,*μ*_(*X*,*Y*) is *false*. That is, each *D*_*i*__*ϕ*,*μ*_(*X*,*Y*) (1≤*i*≤*l*) is *false*. We prove that there does not exist a minmax solution which contains $c {\sqsubseteq } e$ (resp. does not contain $c {\sqsubseteq } e^{\prime }$). Define the solution *S* as

$S = \{ c {\sqsubseteq } x_{i} : \phi (x_{i}) = true, 1 \leq i \leq n \} \cup \{ c {\sqsubseteq } x^{\prime }_{i} : \phi (x_{i}) = false, 1 \leq i \leq n \} \cup \{ c {\sqsubseteq } e\}$. Then *T*∪*S*⊧*M*. Moreover, *S* is subset minimal. Next we show that there exists another subset minimal solution which is more informative than *S*. Define *S*^′^ as

$S^{\prime }= \{ c {\sqsubseteq } y_{i} : \mu (y_{i}) = true, 1 \leq i \leq m \} \cup \{ c {\sqsubseteq } y^{\prime }_{i} : \mu (y_{i}) = false, 1 \leq i \leq m \} \cup \{ c {\sqsubseteq } e^{\prime }\}$. First we show that *T*∪*S*∪*S*^′^ is consistent. From the construction of *T*, we notice that inconsistency can only occur if there is an *x*_*j*_∈*X* (resp. $x^{\prime }_{j} \in X^{\prime }$) such that $c {\sqsubseteq } x_{j} \in S$ (resp. $c {\sqsubseteq } x^{\prime }_{j} \in S$), and $T \cup S \cup S^{\prime } \models c {\sqsubseteq } x_{j}^{\prime }$ (resp. $T \cup S \cup S^{\prime } \models c {\sqsubseteq } x_{j}$) also holds.

Consider any subsumption $\rho = Q {\sqsubseteq } p$ in *T*^′^∪*T*^′′^. Assume *ρ* is regarding the disjunct *D*_*i*_. If for every *z*∈*Q*, $(c {\sqsubseteq } z) \in S \cup S^{\prime }$ holds, then except for one literal (we call it *z*_1_), the truth assignments enable all other literals in *D*_*i*_ to be *true*. Since *D*_*i*__*ϕ*,*μ*_(*X*,*Y*) is *false*, *z*_1_ has to be *false*. If *z*_1_ is a positive literal with the form of *x*, then *x* is assigned as *false* in *ϕ*. Therefore $c {\sqsubseteq } x^{\prime }$ is in *S*. From the construction of *ρ* we obtain that *p* is in fact *x*^′^. Thus $T \cup S \cup S^{\prime } \models c {\sqsubseteq } x^{\prime }$ holds, and *T*∪*S*∪*S*^′^ is consistent. Analogously, if *z*_1_ is a negative literal with the form of ¬*x*, then *x* is assigned as *true* in *ϕ*. Therefore $c {\sqsubseteq } x$ is in *S*. From the construction of *ρ* we obtain that *p* is in fact *x*. Thus $T \cup S \cup S^{\prime } \models c {\sqsubseteq } x$ holds, and *T*∪*S*∪*S*^′^ is consistent.

Now that *T*∪ *S*∪*S*^′^ is consistent, *T*∪*S*∪*S*^′^⊧*S* holds. Further, ($S\, \cup S^{\prime } \setminus \{c {\sqsubseteq } e\}$) is a subset minimal solution. Moreover, it is straightforward to verify that $T \cup (S \cup S^{\prime } \setminus \{c {\sqsubseteq } e\}) \models S$. This concludes the proof.

♣ □

#### Complexity - ${\mathcal {EL}}$

In the following proofs we define the solution *S*_*or*_ as $S_{\textit {or}} = \{ P_{i} ~{\sqsubseteq }~ Q_{i} \mid \forall P_{i}, Q_{i} \in C : Or(P_{i} ~{\sqsubseteq }~ Q_{i}) = true\}$ with the intended meaning that *S*_*or*_ consists of all the subsumptions that are *true* according to the domain expert.

##### General Case

###### Theorem 13.

To decide if ${\mathcal {S}}(T, C, Or, M) \neq \emptyset $ for a given GTAP (*T*,*C*,*Or*,*M*) is in P.

###### *Proof*.

To decide the existence problem, we need to test whether *T*∪*S*_*or*_⊧*M*, and the entailment problem of ${\mathcal {EL}}$ is tractable [6]. Note that *T*∪*S*_*or*_ is consistent, thus if *T*∪*S*_*or*_⊮*M*, then there does not exist a solution.

♣ □

###### Theorem 14.

To decide if a given *ψ* is relevant for a given GTAP (*T*,*C*,*Or*,*M*) is in P.

###### *Proof*.

We assume *Or*(*ψ*) is *true*. Otherwise the relevant problem returns *false*. The problem is equivalent to the existence problem. That is, if there exists a solution *S*, then *S*∪{*ψ*} is also a solution. If there does not exist a solution, then *ψ* is not relevant.

♣ □

###### Theorem 15.

To decide if a given *ψ* is in all the solutions for a given GTAP (*T*,*C*,*Or*,*M*) is in P.

###### *Proof*.

Two entailment tests are called: (1) *T*∪*S*_*or*_⊧*M* and (2) *T*∪(*S*_*or*_∖{*ψ*})⊮*M*. If both (1) and (2) holds, then *ψ* is in every solution. Otherwise, either there does not exist a solution ((1) does not hold), or there is a solution that does not contain *ψ* (*T*∪(*S*_*or*_∖{*ψ*})).

♣ □

##### Subset minimality

###### Theorem 16.

To decide if ${\mathcal {S}}_{\textit {min}}(T, C, Or, M) \neq \emptyset $ for a given GTAP (*T*,*C*,*Or*,*M*) is in P.

###### *Proof*.

The problem is equivalent to the existence problem in general case. Detailed proof see Theorem 3.

♣ □

###### Theorem 17.

To decide if a given *ψ* is min-relevant for a given GTAP (*T*,*C*,*Or*,*M*) is NP-complete.

###### *Proof*.

Hardness follows immediately due to the fact that the min-relevant problem for definite Horn theory problem is NP-complete [65,67]. For the upper bound, we can guess a solution *S* which contains *ψ*, and test whether $S \in {\mathcal {S}}_{\textit {min}}(T, C, Or, M)$. Note that $S \in {\mathcal {S}}_{\textit {min}}(T, C, Or, M)$ iff *T*∪*S*⊧*M* and ∀*h*∈*S*:*T*∪(*S*∖{*h*})⊮*M*. Thus the problem is in NP.

♣ □

###### Theorem 18.

To decide if a given *ψ* is in every minimal solution for a given GTAP (*T*,*C*,*Or*,*M*) is in P.

###### *Proof*.

The upper bound follows the proof in general case in Theorem 15. That is, two entailment tests are called: (1) *T*∪*S*_*or*_⊧*M* and (2) *T*∪(*S*_*or*_∖{*ψ*})⊮*M*. If both (1) and (2) holds, then *ψ* is in every solution, thus also in every solution of ${\mathcal {S}}_{\textit {min}}(T, C, Or, M)$. Otherwise, *S*=*T*∪(*S*_*or*_∖{*ψ*}) is a solution which does not contain *ψ*. Then there is a subset minimal solution *S*^′^⊆*S*. Obviously *S*^′^ does not contain *ψ* as well.

♣ □

##### Semantic maximality

For ${\mathcal {EL}}$ TBox, *S*_*or*_ if *T*∪*S*_*or*_⊧*M* is the most informative solution. Therefore all the decision problems are trivial.

##### Minmax

###### Theorem 19.

To decide if ${\mathcal {S}}_{\textbf {min}}^{max}(T, C, Or, M) \neq \emptyset $ for a given GTAP (*T*,*C*,*Or*,*M*) is in P.

###### *Proof*.

Follows the counterpart in ${\mathcal {EL}^{++}}$, see Theorem 11.

♣ □

###### Theorem 20.

To decide if a given *ψ* is minmax-relevant for a given GTAP (*T*,*C*,*Or*,*M*) is NP-complete.

###### *Proof*.

Hardness follows from the NP-complete complexity of the min-relevance problem. In the following we prove the upper bound. First a subset minimal solution *S* that contains *ψ* can be guessed and tested. Given a solution *S*, we define *closure*(*S*)={*x*:*T*∪*S*⊧*x*}. Next we prove that *S* is minmax optimal iff {∀*h*∈*S*:*T*∪(*S*_*or*_∖*closure*(*S*))∪(*S*∖{*h*})⊮*h*}. If: if ∀*h*∈*S*:*T*∪(*S*_*or*_∖*closure*(*S*))∪(*S*∖{*h*})⊮*h*, then no element from *S* can be derived from outside the closure of *S*. Thus no more informative solution exists. Only if: assume ∃*h*∈*S*:*T*∪(*S*_*or*_∖*closure*(*S*))∪(*S*∖{*h*})⊧*h* holds. Then *S*^′^=(*S*_*or*_∖*closure*(*S*))∪(*S*∖{*h*}) is a solution and *T*∪*S*^′^⊧*S*. We first reduce *S*^′^ to *S*^′′^ such that *T*∪*S*^′′^⊧*S* holds and *S*^′′^ is subset minimal. Next we show that *S*^′′^ is more informative than *S*. Since *S* is subset minimal, *T*∪(*S*∖{*h*})⊮*h* holds. Then from *S*^′′^ we know that there must be an *h*^′^∈*S*^′′^ such that *h*^′^∈(*S*_*or*_∖*closure*(*S*)). Then it follows that *T*∪*S*⊮*h*^′^.

♣ □

###### Theorem 21.

To decide if a given *ψ* is in every minmax solution for a given GTAP (*T*,*C*,*Or*,*M*) is in P.

###### *Proof*.

The upper bound follows the proof in minimal case in Theorem 18.

♣ □

##### Skyline

###### Theorem 22.

To decide if ${\mathcal {S}}_{\textit {min}}^{max}(T, C, Or, M) \neq \emptyset $ for a given GTAP (*T*,*C*,*Or*,*M*) is in P.

###### *Proof*.

The problem is equivalent to the existence problem in general case, thus the upper bound follows immediately.

♣ □

###### Theorem 23.

To decide if a given *ψ* is skyline-relevant for a given GTAP (*T*,*C*,*Or*,*M*) is NP-complete.

###### *Proof*.

The upper bound follows the NP-completeness of the skyline-relevant problem on ${\mathcal {EL}^{++}}$, see Theorem 8. To prove the hardness, we construct a reduction from the relevance problem of the subset minimality for ${\mathcal {EL}}$ as follows. Given a GTAP (*T*,*C*,*Or*,*M*) (denoted as P1) where *T* is a TBox in ${\mathcal {EL}}$, where $M = \{ A~{\sqsubseteq }~B\}$. Note that this simplification does not affect the NP hardness of the problem. We construct another GTAP (*T*^′^,*C*,*Or*,*M*) (denoted as P2), with $T^{\prime } = T \cup \{P_{i} {\sqsubseteq } A, B {\sqsubseteq } Q_{i} | P_{i} {\sqsubseteq } Q_{i} \in S_{\textit {or}}\}$. The intuition of P2 is that if there is a solution *S* such that *T*∪*S*⊧*M*, then both *T*^′^∪*S*⊧*M* and *T*^′^∪*S*⊧*S*_*or*_ hold.

In the following we prove that a given *ψ* is subset minimal relevant to P1 if and only if *ψ* is skyline relevant to P2.

If: Assume *ψ* is skyline relevant to P2. There exists a solution *S*_2_ containing *ψ*, such that there does not exist any solution $S_{2}^{\prime } \subset S_{2}$ and $S_{2}^{\prime }$ is equally informative to *S*_2_. Now we show that *S*_2_ is also a subset minimal solution to P1. First we prove that *T*∪*S*_2_⊧*M*. Assume the opposite: *T*∪*S*_2_⊮*M* holds, then it follows *T*^′^∪*S*_2_⊮*M*, because extending *T* with $\{P_{i} {\sqsubseteq } A, B {\sqsubseteq } Q_{i}\}$ does not result in the subsumption of $A {\sqsubseteq } B$. Assume *S*_2_ is not subset minimal in P1. Then there is another solution $S_{2}^{\prime \prime } \subset S_{2}$, such that $T \cup S_{2}^{\prime \prime } \models M$. Then it follows that $T^{\prime } \cup S_{2}^{\prime \prime } \models M$ and $T^{\prime } \cup S_{2}^{\prime \prime } \models S_{\textit {or}}$. Note that *T*^′^∪*S*_2_⊧*M* and *T*^′^∪*S*_2_⊧*S*_*or*_ also hold, thus *S*_2_ and $S_{2}^{\prime \prime }$ are equally informative in P2, contradiction.

Only if: *ψ* is subset minimal relevant to P1. Then there exist a solution *S*_1_ containing *ψ* and *S*_1_ is a minimal solution. Next we show that *S*_1_ is also a skyline solution to P2. Since *T*⊆*T*^′^, *S*_1_ is also a solution to P2. Since *S*_1_ is minimal to P1, for any subset $S_{1}^{\prime }$ of *S*_1_, we have $T \cup S_{1}^{\prime } \not \models M$. It follows that $T^{\prime } \cup S_{1}^{\prime } \not \models M$, because extending *T* with $\{P_{i} {\sqsubseteq } A, B {\sqsubseteq } Q_{i}\}$ does not result in the subsumption of $A {\sqsubseteq } B$. Thus $S_{1}^{\prime }$ is not a solution to P2. Therefore *S*_1_ is a skyline solution to P2. □

###### Theorem 24.

To decide if a given *ψ* is in every skyline solution for a given GTAP (*T*,*C*,*Or*,*M*) is in P.

###### *Proof*.

Follows Theorem 18.

♣ □

##### Maxmin

###### Theorem 25.

To decide if ${\mathcal {S}}_{\textit {min}}^{\textbf {max}}(T, C, Or, M) \neq \emptyset $ for a given GTAP (*T*,*C*,*Or*,*M*) is in P.

###### *Proof*.

The problem is equivalent to the existence problem in general case, thus the upper bound follows immediately.

♣ □

###### Theorem 26.

To decide if a given *ψ* is maxmin-relevant for a given GTAP (*T*,*C*,*Or*,*M*) is in P.

###### *Proof*.

Follows Theorem 23.

♣ □

###### Theorem 27.

To decide if a given *ψ* is in every maxmin solution for a given GTAP (*T*,*C*,*Or*,*M*) is in P.

###### *Proof*.

Follows Theorem 18.

♣ □
